# Identification of a small-molecule ligand of the epigenetic reader protein Spindlin1 via a versatile screening platform

**DOI:** 10.1093/nar/gkw089

**Published:** 2016-02-17

**Authors:** Tobias Wagner, Holger Greschik, Teresa Burgahn, Karin Schmidtkunz, Anne-Kathrin Schott, Joel McMillan, Lina Baranauskienė, Yan Xiong, Oleg Fedorov, Jian Jin, Udo Oppermann, Daumantas Matulis, Roland Schüle, Manfred Jung

**Affiliations:** 1 Institute of Pharmaceutical Sciences, University of Freiburg, Freiburg 79104, Germany; 2 Department of Urology and Center for Clinical Research, University Freiburg Medical Center, Freiburg 79106, Germany; 3 Department of Biothermodynamics and Drug Design, Institute of Biotechnology, Vilnius University, Vilnius 02241, Lithuania; 4 Department of Structural and Chemical Biology, Department of Oncological Sciences, Department of Pharmacology and Systems Therapeutics, Icahn School of Medicine at Mount Sinai, New York, NY 10029-6574, USA; 5 Structural Genomics Consortium, Nuffield Department of Clinical Medicine, University of Oxford, Target Discovery Institute (TDI), Oxford OX3 7FZ, UK; 6 Structural Genomics Consortium, Botnar Research Center, NIHR Oxford BRU, University of Oxford, Oxford OX3 7LD, UK; 7 German Cancer Consortium (DKTK), Freiburg, Germany; 8 BIOSS Centre of Biological Signalling Studies, University of Freiburg, 79106 Freiburg, Germany; 9 German Cancer Research Centre (DKFZ), Heidelberg, Germany

## Abstract

Epigenetic modifications of histone tails play an essential role in the regulation of eukaryotic transcription. Writer and eraser enzymes establish and maintain the epigenetic code by creating or removing posttranslational marks. Specific binding proteins, called readers, recognize the modifications and mediate epigenetic signalling. Here, we present a versatile assay platform for the investigation of the interaction between methyl lysine readers and their ligands. This can be utilized for the screening of small-molecule inhibitors of such protein–protein interactions and the detailed characterization of the inhibition. Our platform is constructed in a modular way consisting of orthogonal *in vitro* binding assays for ligand screening and verification of initial hits and biophysical, label-free techniques for further kinetic characterization of confirmed ligands. A stability assay for the investigation of target engagement in a cellular context complements the platform. We applied the complete evaluation chain to the Tudor domain containing protein Spindlin1 and established the *in vitro* test systems for the double Tudor domain of the histone demethylase JMJD2C. We finally conducted an exploratory screen for inhibitors of the interaction between Spindlin1 and H3K4me3 and identified A366 as the first nanomolar small-molecule ligand of a Tudor domain containing methyl lysine reader.

## INTRODUCTION

Gene expression is regulated in a hierarchical way by various control-mechanisms. DNA methylation and subsequent oxidation of methylated cytosine residues within DNA and a variety of specific post-translational modifications (PTMs) of histone proteins are the main contributors to epigenetic regulation processes. Covalent PTMs of histone proteins that are created by so-called ‘writer’ enzymes or removed by enzymes termed ‘erasers’ include acetylation, phosphorylation, ubiquitination, ribosylation, sumoylation and methylation (1). These modifications have an intrinsic effect on chromatin structure and activity. Acetylation of lysine residues within histones leads to a loss of the positive charge of the ϵ-ammonium group under physiological conditions which results in a weaker interaction of negatively charged DNA with the histone proteins. Globally, this eventually causes a more accessible chromatin state, also referred to as euchromatin (2). In contrast to this, posttranslational methylation of lysine residues does not alter the charge of the amino acid side chain and there is only a subtle change in size and lipophilicity from the non-methylated to the mono-, di- and trimethylated state of lysines (2). Lysine methylation is associated either with transcriptional activation or repression in a site- and methylation state-dependent manner. Specific patterns of histone marks are recognized by highly specific binding domains of so-called ‘reader’ proteins which are thought to mediate epigenetic signaling. Readers exhibit different functions, like for example recruitment of further chromatin modifying enzymes or transcription factors, stabilization of chromatin complexes or they can possess an intrinsic chromatin modifying activity themselves (3). Methyl lysine reader proteins recognize their interaction partners site-specifically and they can also distinguish between different methylation states (4). Some readers even contain an assembly of different binding domains in one protein and are thus able to interact with more than one PTM at the same time. Hence, reader proteins are therefore often regarded as the ‘interpreters’ of the epigenetic code (5). The methyl lysine readers are categorized into different families by their respective binding domains, namely plant homeodomain (PHD) zinc finger-, WD40 repeat-, ankyrin repeat-, bromo-adjacent homology (BAH)-containing proteins and the Royal family of reader proteins. The latter is further subdivided into Tudor-, chromodomain-, Pro-Trp-Trp-Pro (PWWP)- and malignant brain tumor (MBT) repeat domain-containing proteins (2,6–7). A common feature of all methyl lysine binding domains known so-far is the so-called aromatic cage which is built up by two to four electron-rich aromatic amino acid residues. Cation-π interactions and van der Waals contacts are mainly responsible for the interaction between the methyl lysine site and the aromatic cage of the reading domain.

Misregulation of chromatin often leads to abnormal gene expression patterns which are more frequently linked to human diseases like cancers. Thus, proteins and enzymes that are involved in chromatin regulation processes display interesting targets for drug discovery. In comparison to writers and erasers, readers and their respective binding domains so far have been less intensively pursued as therapeutic targets, especially with regard to methylation. However, aiming at a specific protein–protein interaction could offer an attractive alternative to the inhibition of enzymes that modify histone proteins in a rather promiscuous manner which might subsequently lead to a higher potential of side-effects (2). For this purpose and in order to further investigate the role of methyl lysine reader proteins in the epigenetic interplay and in chromatin regulation, efficient assay systems for the screening of native binding partners (histone and non-histone proteins) and small-molecule ligands for the use as biochemical tool compounds and potential therapeutic agents are highly needed (8).

**Scheme 1.**
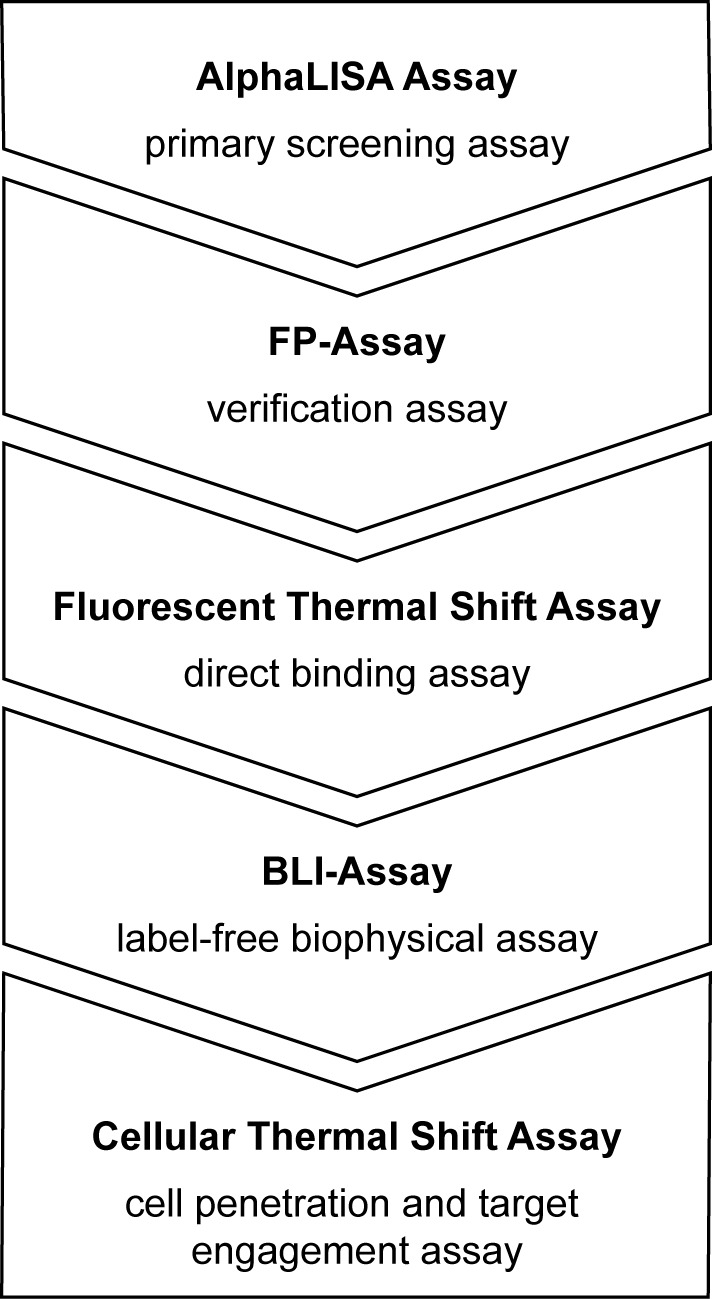
Screening platform for ligands of epigenetic reader proteins.

So far, the screening for new ligands of methyl lysine readers has been rather laborious as the techniques that are frequently utilized either require large amounts of purified protein (like in the case of isothermal titration calorimetry (ITC)(9)) or they need specialized equipment and have limited use for high-throughput screening, e.g. surface plasmon resonance (SPR) or NMR-titration techniques (^1^H-^15^N-HSQC spectra recorded on 500 Hz or 600 Hz NMR spectrometers)(10). Other binding assays based on AlphaScreen (11) and fluorescence polarization (12) have been shown to be useful methods for ligand screening. However, these techniques are not suitable as stand-alone solutions as they are all individually prone to certain assay interferences potentially leading to false positive or false negative screening results. Here, we present a versatile, efficient and powerful platform for the screening of new ligands of methyl lysine reader proteins. This platform consists of a set of orthogonal assays amenable for medium- and high-throughput screening that were set up in a modular fashion. Explicitly, we first established two *in vitro* binding assays based on AlphaLISA technology and fluorescence polarization, respectively. These two *in vitro* assays are complemented by two label-free, biophysical techniques, i.e. a fluorescent thermal shift assay (FTSA) and biolayer interferometry (BLI) that enable the kinetic characterization of initially identified and confirmed ligands. Taken together, the assay modules complement each other with respect to methodology and the risk of potential individual assay interferences can be minimized by combining the different techniques in a screening process. On a cellular level, fluorescence recovery after photobleaching (FRAP) assays employing Green fluorescent Protein (GFP) fusion proteins and traditional immunofluorescence assays are reported as tools for the characterization of intracellular binding events (13). Still, these assays require costly instrumentation (e.g. a laser scanning confocal microscope for FRAP) and labelling techniques that do not work in a completely native background and hence may lead to artefacts. This motivated us to establish a convenient method for the cellular characterization of methyl lysine reader ligands. Therefore, we applied the cellular thermal shift assay (CETSA) methodology (14) for the investigation of target engagement of ligands of reader proteins in a cellular context (see Scheme 1).

As the first and main target of this study we were investigating the methyl lysine reader protein Spindlin1 which recognizes histone H3 with a trimethylated lysine in position 4 (H3K4me3) exclusively by the second of three Tudor-like domains with high affinity (15). Spindlin1 is a potential target for drug development as human *spindlin1* was discovered as an overexpressed gene fragment in ovarian cancer cells. Furthermore, *spindlin1* was shown to be highly expressed in various types of malignant tumor tissues, including non-small-cell lung cancers, ovarian tumors and some hepatic carcinomas (16). Abnormal expression of *spindlin1* causes a cell-cycle delay in metaphase and leads to chromosome instability (17). Furthermore, elevated *spindlin1* expression has been detected in clinical tumor samples (16). Only recently, it was found that reducing Spindlin1 protein levels strongly impairs proliferation and increases apoptosis of liposarcoma cells *in vitro* and in xenograft mouse models underlining the great potential of small-molecule ligands of Spindlin1 for the treatment of cancer (18). As Spindlin1 exemplarily demonstrates, methyl lysine readers offer promising potential as targets for drug development. The well-defined structure of the aromatic cage as the predominant binding site of methyl lysine readers promises relatively high hit rates in screening campaigns as compared to otherwise rather challenging attempts to interrupt protein–protein interactions. However, no small-molecule ligands of Spindlin1 that might act as inhibitors of the interaction between the reader domain and its native binding partner H3K4me3 have been reported until today. This motivated us to apply our assay platform to the screening of such small-molecule ligands of Spindlin1 and we present the first successful identification of a potent Spindlin1 ligand.

## MATERIALS AND METHODS

### Peptides

All peptides were custom synthesized by Peptide Specialty Laboratories GmbH (Heidelberg, Germany). A biotinylated peptide derived from histone H3 containing a trimethylated lysine sidechain in position 4 (H_2_N-ARTK(me3)QTARKSTGGKAPRKQLATK(biotin)-COOH; biotin linked via the sidechain of the C-terminal lysine 23) was used for the AlphaLISA assay and for BLI measurements and named biotin-H3(1–23)K4me3. A fluorescein labeled peptide derived from histone H3 (ARTK(me3)QTARKSTGGK, mixed isomers of 5-(and-6)-carboxyfluorescein coupled to K14 side chain) was used as a probe for the fluorescence polarization based assay and named FL-H3K4me3. An unlabeled form of the histone H3 derived peptide (H_2_N-ARTK(me3)QTARKSTG-COOH) was used as reference competitor in all assays and named H3(1–12)K4me3. In CETSA we used an unmethylated variant of the histone H3 derived peptide (H_2_N-ARTKQTARKSTGGKAPRKQLA-GGK(biotin)-COOH) for control experiments which was named H3(1–21)K4me0.

### Expression and purification of recombinant Spindlin1 and JMJD2C protein

For expression in *Escherichia coli*, the human *spindlin1* cDNA (encoding amino acids 49–262) and the murine JMJD2C cDNA (encoding amino acids 874–988) were subcloned into a modified pET15b expression plasmid harboring a minimal, non-cleavable hexahistidine (His) tag. Recombinant proteins were expressed in *E.coli* BL21(DE3) codon plus (Stratagene). Main cultures of 1.8 l terrific broth medium (Merck) supplemented with 0.4% glycerol and 100 μg/ml ampicillin in 5 l flasks were inoculated with 20 ml start culture and grown at 37°C until an OD_600_ of ∼0.8 was reached. Cultures were then cooled to 16°C, induced with 0.1 mM IPTG (Spindlin1) or 0.5 mM IPTG (JMJD2C) and cultivated for additional 16 h. Cells were harvested by centrifugation (10 min, 5000 *xg*, 4°C), pellets were washed with 0.9% NaCl solution and then stored at −20°C.

For purification of His-Spindlin1(49–262) protein, *E. coli* pellets were resuspended in buffer (∼5 ml per 1 g of pellet) containing 20 mM Tris/HCl (pH 8.0), 300 mM NaCl, 5 mM imidazole and disrupted by sonication. After centrifugation (15 min, 20000 x *g*, 4°C) the supernatant was passed through a standard paper filter (Schleicher & Schuell) and incubated for 2 h at 4°C with Talon beads (Clontech), which were equilibrated with buffer containing 20 mM Tris/HCl (pH 8.0), 300 mM NaCl, 5 mM imidazole. Beads were washed twice with equilibration buffer and then collected in Poly-Prep chromatography columns (Bio-Rad). His-Spindlin1(49–262) was eluted from Talon beads with buffer containing 20 mM Tris/HCl (pH 8.0), 100 mM NaCl, 150 mM imidazole and further purified by gel filtration using a superdex S75(16/60) column (GE Healthcare) in buffer containing 20 mM Tris/HCl (pH 8.0), 100 mM NaCl. Peak fractions with a molecular weight of about 25 kDa were pooled and concentrated to ∼16 mg/ml using Vivaspin Turbo 4 concentrators (10 000 MWCO, Sartorius). Protein aliquots were stored at −80°C. The same strategy involving Talon resin and gel filtration was used for the purification of His-JMJD2C(874–988) proteins, with the exception that in all buffers 20 mM Tris (pH 8.0) was replaced with 50 mM Tris (pH 7.5).

### Generation of anti-Spindlin1 antibody

The generation of a polyclonal rabbit antibody directed against glutathione-S-transferase-tagged Spindlin1 (amino acids 183–229) was described previously (18).

### Cell culture

HL-60 cells were cultured in RPMI1640 medium supplemented with 10% Fetal calf serum (FCS), penicillin/streptomycin and glutamine in an incubation chamber (Heraeus CO_2_-Auto-Zero) at 37°C with 5% CO_2_. T-175 cell culture flasks were used for maintaining cells and T-75 flasks (Sarstedt, Nümbrecht, Germany) were used for CETSA experiments (see section below). Cells were maintained at 0.5–1.0 × 10^6^ cells/ml and split 1:2 to 1:5 every 1–2 days. For CETSA experiments cells were raised to a density of 1.5–2.0 × 10^6^ cells/ml and a viability of >98% was ensured by splitting the cells in fresh medium at least 24 h previous to intended experiments. Determination of cell numbers and viability was conducted with Luna™fl Dual Fluorescence Cell Counter using Luna™ Cell Counting Slides (Biozym Scientific GmbH, Hessisch Oldendorf, Germany).

### SDS-PAGE and Western blot analysis

A total of 12% polyacrylamide gels (mini format) were freshly prepared following standard procedures and used to separate proteins in samples with the help of Mini-PROTEAN^®^ Tetra Systems equipped with a PowerPac™ Basic (Bio-Rad, Hercules, USA). ColorPlus™ Prestained Protein Ladder (cat. no. P7711, New England BioLabs Inc., Ipswich, USA) was used for control of molecular weights of separated proteins. Loading buffer was added to the samples and the resulting mixture was heated to 70°C for 10 min in a heating block. For CETSA, 13 μl of cell lysate samples were loaded onto the gel in the order of increasing incubation temperatures. SDS-PAGE was performed by applying 160 V for ∼75 min under standard buffer conditions. Gels were then carefully rinsed with water before proteins were transferred to 0.2 μm mini format nitrocellulose membranes (cat. no. 170–4158, Bio-Rad, Hercules, USA) using the Trans-Blot^®^Turbo™ Transfer System (Bio-Rad, Hercules, USA). The membranes were blocked for 60 min with blocking buffer (5% milk powder in Tris buffered Saline (TBS) buffer supplemented with 0.1% Tween-20). For immunoblotting, membranes were incubated at 4°C overnight with anti-Spin1 antibody (rabbit) as primary antibody diluted 1:500 in blocking buffer. Goat anti-rabbit IgG HRP (A0545, Sigma) was employed as secondary antibody diluted 1:1000 in blocking buffer in a 2 h incubation step at room temperature. After each step membranes were washed for 3 × 10 min in TBS buffer supplemented with 0.1% Tween-20. The membranes were developed subsequently using Clarity™ Western ECL Substrate (Bio-Rad, Hercules, USA) according to the manufacturer's recommendations. Chemiluminescence intensities were detected and quantified using a Fusion SL™ imaging system (Viber Lourmat, Eberhardzell, Germany). Note that for CETSA, the exposure should lead to a saturated image within seconds in order to obtain representative and similar band intensities. Melting curves were generated by relating the band intensities of each sample to the band intensity obtained from the sample heated with the lowest temperature and plotting the relative band intensities against the respective temperature in GraphPad Prism software (version 4; La Jolla, USA). Curve fitting was conducted by applying a Boltzmann sigmoidal dose-response model.

### AlphaLISA assay

#### Peptide–protein binding matrix titration

Dilution series of recombinant His-Spindlin1(49–262) and of biotin-H3(1–23)K4me3 peptide were prepared in assay buffer (25 mM Hepes (pH 7.5); 100 mM NaCl; 1 mg/ml BSA; 0.05% CHAPS) covering a concentration range of 2 nM up to 1 μM, respectively. Both binding partners were cross-titrated on a 384-well AlphaPlate (PerkinElmer, Waltham, USA) by combining 10 μl of each binding partner per well resulting in a binding matrix with a final concentration range of both binding partners of 1 to 500 nM in a volume of 20 μl. The plate was sealed with an adhesive film (TopSeal^®^-A, PerkinElmer) and gently shaken for 30 min at 25°C. A dilution of nickel chelate AlphaLISA^®^ acceptor beads (PerkinElmer, cat. #AL108M) and AlphaScreen^®^ streptavidin donor beads (PerkinElmer, cat. #6760002) was prepared in assay buffer to provide a concentration of 0.083 mg/ml (1:60 dilution of 5 mg/ml stock). Five microliters of this mixture was added to each well of the matrix resulting in a final assay volume of 25 μl and a final Alpha bead concentration of 0.017 mg/ml (1:300 dilution of 5 mg/ml stock). Note that all steps involving handling with AlphaScreen^®^ donor beads have to be performed under subdued light as the beads are light sensitive. The plate was further incubated for 60 min at 25°C. The plate was spun down at 300 x *g* for 2 min before measurement of the Alpha signal on a 2102 EnVision^®^ Multilabel reader (PerkinElmer) in Alpha-mode. The resulting Alpha-signals were blank-corrected and then plotted against His-Spindlin1 and H3K4me3-biotin concentrations, respectively, using GraphPad Prism.

Z′-factors (19) to estimate the assay quality were calculated using the following equation:

(1) \begin{equation*} Z^\prime = 1 - \frac{{3 \times SD_{max} + 3 \times SD_{min} }}{{\left| {\mu _{max} - \mu _{min} } \right|}} \end{equation*}

where SD_max_ and SD_min_ are standard deviations of the positive- and negative control measurements, respectively, and μ_max_ and μ_min_ are the mean of the respective positive- and negative signal controls.

#### AlphaLISA displacement assay

Recombinant His-Spindlin1(49–262) and biotin-H3(1–23)K4me3 were mixed in assay buffer at fixed concentrations of 30 and 60 nM, respectively, and preincubated for 10 min at room temperature. Stock solutions of analytes (10 mM) were prepared in DMSO. For IC_50_ determination experiments, serial 1:1 dilutions of analytes in assay buffer were performed (final concentrations ranging from 0.1 to 200 μM) while maintaining the DMSO concentration in all dilutions at 4% (or DMSO content of the solution containing the highest amount of analyte stock solution in buffer, i.e. the highest compound concentration in the assay). Ten microliters 2x ligand solution were dispensed onto a 384 well AlphaPlate^TM^ (PerkinElmer) in triplicates. Ten microliters of the His-Spindlin1(49–262)/biotin-H3(1–23)K4me3 solution were added to each well containing ligand solution (A_I_) or 10 μl of 4% DMSO in assay buffer (A_pos_, positive control). As negative control (A_neg_), 20 μl of 2% DMSO in assay buffer were added to the microplate. The final concentrations were 15 nM His-Spindlin1(49–262) and 30 nM biotin-H3(1–23)K4me3 in a volume of 20 μl. The sealed microplate was then incubated at 25°C for 60 min (or as indicated in the text). Streptavidin coated AlphaScreen donor beads and nickel chelate functionalized AlphaLISA acceptor beads were diluted and mixed in assay buffer to obtain 0.08 mg/ml of each bead species. Five microliters of mixed beads were added to each well on the microplate leading to a final bead concentration of 0.017 mg/ml per well. A total of 25 μl of 1.6% DMSO in assay buffer were dispensed into 16 wells as blank controls. The sealed plate was further incubated at room temperature for 60 min (or as indicated in the text) in the dark with respect to light sensitivity of the donor beads. The readout was recorded using an EnVision^TM^ plate reader (PerkinElmer) in Alpha-mode. Averages of all controls were calculated after subtraction of blank values from the raw data. Inhibition values (I) were calculated using the following equation:

(2) \begin{equation*} I = 100 \times \left( {1 - \left( {\frac{{A_I - A_{neg} }}{{A_{pos} - A_{neg} }}} \right)} \right)(\% ) \end{equation*}

GraphPad Prism was used for visualization and calculation of IC_50_ values by plotting inhibition values against logarithmic compound concentrations and applying a sigmoidal dose-response fit (variable slope).

The setup of the Alpha assay for the double Tudor domain of JMJD2C (His-JMJD2C (874–988)) was realized by following the same optimization route as described above. His-JMJD2C (874–988) was incubated with biotin-H3(1–23)K4me3 peptide at final concentrations of 300 nM and 25 nM, respectively. Final donor and acceptor bead concentrations were 0.02 mg/ml. All other assay conditions were as described above.

#### AlphaLISA TruHits verification assay

The TruHits verification assay kit (PerkinElmer, cat. #AL900D) contains AlphaLISA^®^ BSA-biotin acceptor beads and streptavidin functionalized AlphaScreen donor beads which interact together to generate an Alpha signal without addition of any further interaction partners. A bead premix was prepared containing both bead species diluted in assay buffer (0.019 mg/ml of each bead type leading to a concentration of 0.011 mg/ml after addition to sample dilutions in a final assay volume of 25 μl) which was preincubated at room temperature for 30 min. A total of 10 μl of test compounds was dispensed onto a 384-well Alpha plate in duplicates and the preincubated bead premix (15 μl) was subsequently added. Thus, the test compounds are brought to similar concentrations as in the AlphaLISA assay. The microplate was sealed and incubated at room temperature for 10 min. After spinning down the plate for 2 min at 300 x *g*. Alpha signals were measured using the EnVision™ plate reader as described above. Potential quenching effects of the Alpha signal were determined by comparing the TruHits signal of positive controls (A_pos_, only TruHits bead premix and buffer) to the signal of the compound-bead mixtures (A_I_) after subtraction of the blank values (only buffer and no bead mixture). The amount of Alpha signal quenching (Q) was calculated by the following equation:

(3) \begin{equation*} Q = 100 \times \left( {1 - \frac{{A_I }}{{A_{pos} }}} \right)(\% ) \end{equation*}

### Fluorescence polarization assay

#### Recording of total fluorescence intensities of the probe

Serial dilutions of FL-H3K4me3 were made in assay buffer (containing 25 mM Hepes (pH 7.5), 100 mM NaCl, 1 mg/ml BSA and 0.05% CHAPS) covering a range of 0.06–750 nM and dispensed on a black 384-well non-binding microplate (Greiner Bio-One GmbH, Frickenhausen, Germany) in triplicates (20 μl). Blank controls contained only buffer (20 μl).

Fluorescence intensities were recorded with an EnVision™ plate reader (PerkinElmer) using the following settings: mirror – FITC FP, excitation filter – FITC FP 480 nm, emission filter 1 – FITC FP p-pol 535 nm, emission filter 2 – FITC FP s-pol 535 nm. Total fluorescence intensities were calculated as $2 \times I_P + I_S$. The resulting values were plotted against the respective probe concentrations with GraphPad Prism (version 4) and fitted with linear regression.

#### Binding curves and K_D_ determination

A 2x concentrated serial dilution series of His-Spindlin1(49–262) in buffer was made covering a total concentration range of 0.06 to 750 nM (final concentrations). Ten microliters of the 2x Spindlin1 dilutions and 10 μl of 20 nM FL-H3K4me3 were dispensed on a black 386-well non-binding microplate (Greiner Bio-One) resulting in a final assay volume of 20 μl per well. Fluorescence polarization (P_M_) was measured from these wells after incubation for 60 min at 25°C with the help of an EnVision^TM^ plate reader (PerkinElmer) using the following settings: mirror – FITC FP, excitation filter – FITC FP 480 nm, emission filter 1 – FITC FP p-pol 535 nm, emission filter 2 – FITC FP s-pol 535 nm. In general, fluorescence polarization (P) values were determined as $\frac{{(I_S - G \times I_P )}}{{(I_S + G \times I_P )}} \times 1000(mP)$, where I_S_ is the fluorescence intensity measured in the s-plane and I_P_ is the fluorescence intensity measured in the p-plane, both blank corrected. G is the grating factor which is an instrument specific constant. Spindlin1 dilutions were also incubated without probe to correct any intrinsic background fluorescence (I_┴_ and I_║_) arising from the protein and not from the probe. Additionally, all Spindlin1 dilutions were combined with 10 nM FL-H3K4me3 (final concentration) plus a saturating concentration (10 μM, final concentration) of unlabeled H3(1–12)K4me3. The so obtained mP values (P_I_) were used to calculate the contribution of unspecific binding (P_NS_) to P_M_. Fluorescence polarization measurements of 10 nM FL-H3K4me3 (P_D*_) and of 10 nM FL-H3K4me3 combined with 750 nM Spindlin1 (final concentrations) provided the mP values for a situation where the probe is free in solution, or bound to Spindlin1 in a saturated manner (P_D*R_), respectively. P_D*_ was further used to set the G-factor (0.91) for our plate reader to obtain a value of ∼25 mP. The obtained values were used to calculate the specific binding of FL-H3K4me3 to every Spindlin1 concentration as follows:

First, a background correction of P_M_ was performed by subtracting fluorescence intensities obtained from the corresponding protein concentrations alone (I_┴_ and I_║_). Next, P_NS_ was calculated for each protein concentration from P_I_ and P_D*_ with $P_{NS} = (P_I - P_{D*} ) \times (1 - F_B )$, where F_B_ is the bound probe fraction. F_B_ was in turn calculated by $F_B = \frac{{P_M - P_{D*} }}{{P_{D*R} - P_{D*} }}$. In the next step, P_S_ was determined from P_M_ and P_NS_ as $P_S = P_M - P_{NS}$. Finally, P_S_ was plotted to a binding curve with GraphPad Prism by using a one-site binding (hyperbola) equation:

(4) \begin{equation*} Y = \frac{{B_{max} \times [R]}}{{K_D + [R]}} \end{equation*}

where *B_max_* is the maximal binding.

#### Fluorescence polarization displacement assay

A 2x concentrated pool solution containing 200 nM His-Spindlin1(49–262) and 20 nM FL-H3K4me3 was prepared in assay buffer. For negative controls a 2x concentrated solution only containing 20 nM FL-H3K4me3 was prepared. For IC_50_ determination experiments, 1:1 ligand dilution series (2x concentrated) were prepared as described above (see AlphaLISA assay). Ten microliters of each ligand solution was dispensed onto a black 384-well non-binding microplate (Greiner) in triplicates. Ten microliters of an appropriate DMSO solution in assay buffer were dispensed into 6 wells for positive controls and into another 6 wells for negative controls. Ten microliters of the Spindlin1/FL-H3K4me3-premix were added to each well containing the ligand solutions (P_I_) and the positive controls (P_pos_). Ten microliters of the FL-H3K4me3 solution were added to the negative control wells (P_neg_). This lead to final concentrations of 100 nM and 10 nM of Spindlin1 and FL-H3K4me3, respectively, in a final assay volume of 20 μl. Six wells contained 20 μL of DMSO solutions in assay buffer to generate blank controls. The microplate was then incubated for 60 min at 25°C.

Inhibition values (I) were calculated using the following equation:

(5) \begin{equation*} I = 100 \times \left( {1 - \left( {\frac{{P_I - P_{neg} }}{{P_{pos} - P_{neg} }}} \right)} \right)(\% ) \end{equation*}

GraphPad Prism was used for visualization and calculation of IC_50_ values by plotting inhibition values against logarithmic compound concentrations and applying a sigmoidal dose-response fit (variable slope).

The setup of the FP assay for the double Tudor domain of JMJD2C (His-JMJD2C (874–988)) was realized by following the same optimization route as described for Spindlin1. In the final displacement assay, His-JMJD2C (874–988) was incubated with FL-H3K4me3 peptide at final concentrations of 10 μM and 15 nM, respectively. All other assay conditions were as described above.

### Fluorescent thermal shift assay

#### Optimization of SYPRO^®^ Orange and reader protein concentrations

On a FrameStar^®^ 96 PCR plate (4titude, Surrey, UK) 10 μl of 4 serial dilutions of SYPRO^®^ Orange (Invitrogen, purchasable stock solution 5000x in DMSO) in buffer (containing 100 mM Hepes (pH 7.5) and 150 mM NaCl) were dispensed and combined with 10 μl of varying His-Spindlin1(49–262) concentrations resulting in a total assay volume of 20 μl per well. Final concentrations were 1.25x, 2.5x, 5x and 10x of SYPRO Orange and 0.0125 mg/ml, 0.025 mg/ml, 0.05 mg/ml, 0.1 mg/ml, 0.2 mg/ml, 0.4 mg/ml and 0.8 mg/ml of Spindlin1. Additionally, negative controls were dispensed on the plate containing 20 μl of the SYPRO Orange solutions (1.25x, 2.5x, 5x and 10x) in buffer and no protein. Blank wells contained 20 μl of buffer. Every combination was added to the plate in duplicates. The plate was sealed with qPCR Seal adhesive sheets (4titude, Surrey, UK) and preincubated at 25°C for 15 min.

Next, the plate was exposed to a temperature gradient program (25–95°C, 0.5°C intervals) and fluorescence intensity was measured after 15 s at each heating step in a MyiQ™ Single Color Real-Time PCR Detection System (filter setting for SYPRO Orange fluorescence measurements: 485/20 nm for excitation, 530/30 nm for emission) equipped with an iCycler (Bio-Rad, Hercules, USA). The fluorescence intensities were plotted against their corresponding temperatures with the help of Bio-Rad CFX Manager (version 2.1) and exported to Microsoft Excel. For calculations of T_m_ values the DSF analysis tool (Excel datasheet and GraphPad preset) was used, provided by Niesen *et al*. (ftp://ftp.sgc.ox.ac.uk/pub/biophysics) (20). GraphPad Prism was used for visualization of the results.

#### Ligand screening setup of the FTSA

A mixture containing 0.1 mg/ml (equal to 3.88 μM) His-Spindlin1(49–262) and 5x SYPRO Orange (final concentrations) was preincubated at 25°C for 30 min with ligand (L) or with buffer (P, positive control). Negative controls contained 5x SYPRO Orange diluted in buffer and no protein. Blank wells contained 20 μl of buffer. If ligand dilutions have been prepared from DMSO stock solutions, the DMSO content was maintained at equal levels among all samples. Plates were sealed with qPCR Seal adhesive sheets (4titude) and subsequently treated as described above. T_m_ shifts (ΔT_m_) were calculated by subtracting T_m_ of positive controls from T_m_ of ligand treated samples. Similar strategies have been pursued for ligand screening approaches of JMJD2C-Td using final concentrations of 0.4 mg/ml (equal to 27 μM) His-JMJD2C(874–988) and 5x SYPRO Orange.

#### K_D_ determination of the H3(1–12)K4me3-Reader interaction

Transition midpoint values of T_m_ resulting from a serial dilution series of H3(1–12)K4me3-peptide incubated with 0.1 mg/ml His-Spindlin1(49–262) (or 0.4 mg/ml His-JMJD2C(874–988)) and 5x SYPRO Orange (final concentrations) were compared to reference values obtained from mixtures of 0.1 mg/ml Spindlin1 (or 0.4 mg/ml JMJD2C) and 5x SYPRO Orange without ligand. Each condition was prepared on a 96-well PCR plate in triplicates and FTSA was conducted as described above. The equillibrium binding constants (*K_D_*s) were then determined by the simulation and regression of the ligand dosing *T_m_*s shown in Figures 3C and 7E to the equation describing the theoretical function of the melting temperature shift dependence on ligand concentration as previously described (21–23).

### BLI assay

#### K_D_ determination of the interaction between His-Spindlin1(49–262) and biotin-H3(1–23)K3me3

Biolayer Interferometry was measured using the BLItz™ system (Pall, Port Washington, USA) in combination with streptavidin functionalized biosensors (ForteBio). Loading of biosensors was conducted by exposing samples containing 0.01 mg/ml biotin-H3(1–23)K4me3 to previously hydrated biosensor tips (100 mM Tris (pH 7.5), 150 mM NaCl and 0.05% CHAPS) for 15 s. Baselines were recorded for 30 s from buffer in both, drop- and tube position.

Association of samples containing increasing amounts His-Spindlin1(49–262) to loaded biosensor tips was recorded for 120 s from drop-holder position (sample size 4 μl). Dissociation was measured by dipping the biosensor tip into a tube filled with buffer for 120 s. Reference measurements were conducted by using buffer instead of Spindlin1-samples. The sample-sensorgrams were corrected by subtracting the reference curve. Global 1:1 fitting of association- and dissociation curves with BLItz Pro 1.2 software revealed *k_a_, k_d_* and *K_D_* binding constants. At least six different concentrations of His-Spindlin1 have been measured and globally fitted for accurate *K_D_* determinations of the Spindlin1/biotin-H3K4me3 interaction.

#### Displacement assay setup for the screening of Spindlin1 ligands

Samples were prepared containing a fixed His-Spindlin1(49–262) concentration of 200 nM and increasing amounts of potential Spindlin1 ligands (I). Positive control samples (P) contained solutions of 200 nM His-Spindlin1 in buffer. The sample solutions were preincubated in PCR-tubes for 30 min at 25°C previous to BLI measurements. Reference curves were recorded as mentioned above. Binding of ligands to Spindlin1 in sample solutions lead to reduced free amounts of Spindlin1 available for binding to loaded biosensor tips. Thus, binding signals of ligand containing Spindlin1 samples were decreased compared to positive controls. Quantitation of this effect was realized by setting report points at 5 s previous to the end of the association phase. The resulting binding values were used for calculation of inhibitory effects by relating the binding value of ligand containing samples to the binding value of the positive control.

Alternatively, competitive effects of Spindlin1 ligands were assessed by measuring the change in dissociation rate (*k_d_*) after saturating biotin-H3(1–23)K4me3-loaded biosensors with samples containing 200 nM His-Spindlin1 for 100 s. Dissociation was then recorded for 160 s by immersing the biosensors into tubes filled with 250 μl buffer (positive control) or 250 μl of samples containing increasing amounts of ligand. Presence of competitive ligands of Spindlin1 resulted in an increased dissociation rate of Spindlin1 from biotin-H3(1–23)K4me3-loaded biosensors which was calculated by local 1:1 fittings with BLItz Pro 1.2 software, based on a theoretical Spindlin1 concentration of 200 nM.

#### Regeneration of biosensors loaded with biotin-H3K4me3 peptide

Redundant amounts of His-Spindlin1(49–262) were removed from biosensors by applying a protocol comprising three cycles of washing with 10 mM glycine (pH 1.5) solution and with buffer in an alternating fashion (each for 20 s in tubes). Thus, biotin-H3(1–23)K4me3-loaded biosensors could be re-used up to 10 times.

### CETSA

HL-60 suspension cells were raised to a density of ∼2 million cells/ml in RPMI1640 medium. A number of ∼20 million cells is needed per CETSA-curve.

- Cells were treated with compound or DMSO as control at 37°C with 5% CO_2_ for 60 min and gently shaken every 10 min. Cell-viability was evaluated immediately after addition of compound/DMSO and after the incubation step.
- Cells were spun down at 500 *xg* for 5 min at room temperature. Medium was carefully removed and cells were resuspended in PBS buffer. Cells were spun down one more time at 500 x *g* for 5 min at room temperature. Phosphate buffered saline (PBS) was carefully removed again and the cell pellet was resuspended in PBS buffer supplemented with protease inhibitors to obtain a cell density of 4 × 10^7^ cells/ml.
- The suspension was separated into 13 aliquots à 45 μl in PCR-tubes and kept at room temperature. Tubes were loaded to the heating block of a peqSTAR 96X Universal Gradient Thermocycler (Peqlab, Erlangen, Germany) at 25°C. Samples were heated to their desired temperatures in parallel by applying a temperature gradient covering a range between 40°C and 76°C (intervals of 3°C). The respective temperatures were maintained for 3 min before the samples were cooled and maintained at 25°C for 3 min. Next, the tubes were immediately shock-frozen in liquid nitrogen.
- Cells were lysed by three alternating thaw-freeze cycles in a heating block (25°C) and in liquid nitrogen, respectively. After each step the suspensions were briefly vortexed and spun down.
- The resulting suspensions were centrifuged with 20 000 *xg* for 20 min at 4°C. For the following steps the lysates were kept on ice. Twenty five microliters of the supernatants of each sample were carefully transferred to reaction tubes without touching or disturbing the pellets.
- Quantitation of the soluble Spindlin1 fraction was realized by SDS-PAGE and Western-blot analysis as described above. For generation of melting curves in GraphPad Prism, data were first normalized by setting the highest and lowest value within each Western-blot membrane to 100 and 0%, respectively. Data were then fitted to obtain apparent T_agg_ values using the Boltzmann Sigmoid equation.

CETSA was performed with lysates by using the following protocol order with changes as indicated in parenthesis: ii, iv., i. (Lysates were treated with compound or DMSO as control at 37°C for 60 min in reaction tubes whilst gently shaking), iii., v., vi.

### Compounds for screening

Compounds that were screened for binding to His-Spindlin1(49–262) were purchased from commercial sources (Chembridge, Princeton, Enamine, Tocris) and were used without further purification. Compounds synthesized in-house were at least 95% pure as determined by HPLC. Compound YX-11–102 was synthesized according to the procedures described in the Supplementary information.

## RESULTS

### AlphaLISA assay

#### Principle and validation

**Figure 1.**
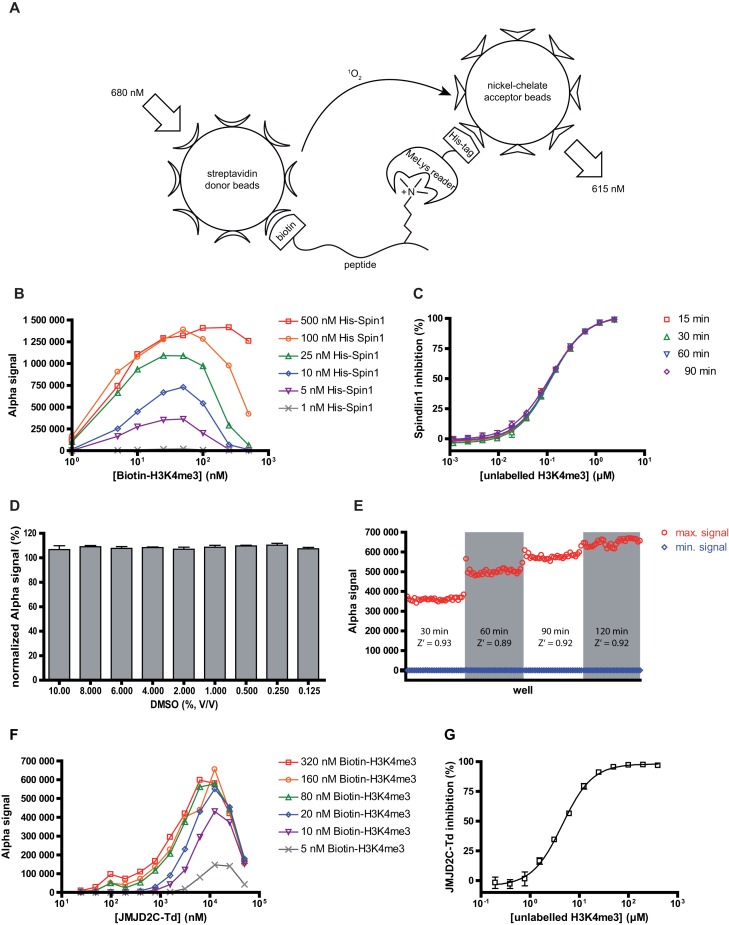
Setup of the AlphaLISA based ligand screening assay. (**A**) Principle of the detection of the interaction between methyl lysine reader proteins and their native ligand-derived and biotinylated peptide. (**B**) Hook-point detection via matrix cross-titration of a panel of His-Spindlin1 concentrations against a dilution series of biotin-H3(1–23)K4me3. Data represent single measurements. (**C**) H3(1–12)K4me3 dose-response inhibition curve of Spindlin1. Unlabeled H3K4me3 peptide competed with 30 nM biotin-H3(1–23)K4me3 for binding to 15 nM His-Spindlin1. The incubation time of the competitor and the His-Spindlin1/biotin-H3K4me3 mixture was varied between 15 and 90 min. Data represent the mean and standard deviation of triplicate measurements. (**D**) dimethyl sulfoxide (DMSO) tolerance of the assay. A panel of DMSO concentrations was exposed to the assay mixture and the resulting influence on the assay window was plotted. Data were normalized against maximum control measurements containing no DMSO and represent the mean and standard deviation of triplicate measurements. (**E**) Evaluation of bead incubation time and Z’-factor determination. The bead incubation step was performed comparing four different time periods (30, 60, 90 and 120 min). Each experiment included 32 positive controls (max. signal) and 32 negative controls (min. signal) and was conducted on separate 384-well plates. For the final assay protocol a bead incubation time of 90 min (S/B = 1200) was adopted. (**F**) Transfer of the AlphaLISA assay setup to the double Tudor domain of JMJD2C. A matrix cross-titration of a panel of JMJD2C-Td concentrations against a dilution series of biotin-H3(1–23)K4me3 revealed the optimal concentrations of the binding partners for subsequent experiments. Data represent single measurements. (**G**) Dose-response inhibition curve of unlabeled H3(1–12)K4me3 which competed with 200 nM biotin-H3(1–23)K4me3 for binding to 5 μM JMJD2C-Td. Data represent the mean and standard deviation of duplicate measurements.

Alpha (amplified luminescent proximity homogeneous assay) technology is making use of colloidal bead suspensions consisting of donor- and acceptor beads which produce a chemoluminescent signal after excitation at a certain wavelength if they are brought into close proximity. Donor beads contain a photosensitizer which upon excitation converts ambient oxygen to singlet oxygen. Singlet oxygen has a half-life of about 4 μs, which allows it to diffuse ∼200 nm in solution. Only if an acceptor bead is within that distance to the excited donor bead, a luminescence signal is generated through energy transfer of singlet oxygen to the acceptor bead. Through this, an europium chelate, which is also present on the acceptor bead, is directly excited creating light of a narrow wavelength bandwidth centered around 615 nm (24). Donor- and acceptor beads are available with different functionalizations. We made use of the 6xHis-tag present on our recombinant Spindlin1 protein by coupling it to Ni-NTA-coated acceptor beads. Streptavidin-coated donor beads were used to capture a biotinylated peptide derived from the endogenous ligand of Spindlin1, namely histone protein H3 trimethylated at position 4 (H3K4me3; amino acids 1–23). Through interaction of His-Spindlin1 with its endogenous ligand the two bead types were brought into an adequate proximity so that an assay signal could be generated. Thus, by measuring the strength of the Alpha signal the portion of biotin-H3(1–23)K4me3 bound to Spindlin1 could be determined. A schematic view of our AlphaLISA assay is illustrated in Figure 1A.

#### Spindlin1 AlphaLISA assay optimization

In a pretest we evaluated the optimal acceptor- and donor bead concentrations for the Alpha assay using a biotin-6xHis peptide which interacts with both bead species bringing them into proximity to generate an Alpha signal. Besides the manufacturer's recommendation of 25 μg/ml (final concentration) of both bead types, we tested several dilutions of a 1:1 acceptor- and donor bead mixture (data not shown). We observed an appropriate assay window at a concentration of 17 μg/ml for both, acceptor- and donor beads (1/300 dilution of a 5 mg/ml stock-suspension), in the final assay volume. Next, we were scouting for the optimal assay concentrations of both, His-Spindlin1 and its ligand biotin-H3(1–23)K4me3. Therefore, we conducted a matrix cross-titration of His-Spindlin1 and biotin-H3(1–23)K4me3 on a microplate. After incubation of the plate and addition of Ni-NTA acceptor beads and streptavidin donor beads the Alpha signals were collected and plotted against the concentrations of His-Spindlin1 and biotin-H3K4me3 concentrations, respectively. The resulting titration curves showed the so-called hook-effect which is typically observed when the concentration of analytes exceeds the binding capacity of the respective bead-type leading to dilution effects and non-statistical distribution of bead-bound and unbound Spindlin1/biotin-H3K4me3 complexes. The characteristic shape of such a curve shows an initial increase of the Alpha signal as the concentration of one binding partner increases followed by a decrease of the signal after the concentration of the binding partner surpasses the binding capacity of the respective Alpha bead. In our experiment we observed a significant saturation and dilution effect for the streptavidin coated donor beads at concentrations of the biotinylated peptide exceeding 50 nM (final concentration). For the acceptor beads no hook-effect could be observed with His-Spindlin1 up to concentrations equal to 500 nM of the protein (see Figure 1B). According to the manufacturer this apparent discrepancy in binding capacity of the streptavidin donor beads and the Ni-NTA acceptor beads is due to the fact that the 6xHis-Ni-NTA interaction is weaker compared to the biotin-streptavidin interaction. Thus, more 6xHis-tagged protein dissociates from the acceptor beads at equilibrium which is why the hook-point is typically reached at higher protein concentrations for the Ni-NTA acceptor beads compared to the streptavidin donor beads. As a result of this experiment we chose a His-Spindlin1 concentration of 15 nM for subsequent experiments and a biotin-H3(1–23)K4me3 concentration of 30 nM, respectively. These concentrations display a compromise between an appropriate assay window and a reliable prevention of the hook-effect.

As our goal was to use this assay for the screening of new Spindlin1 ligands, we measured the displacement of biotin-H3(1–23)K4me3 from a preformed biotin-H3K4me3/Spindlin1 complex by disturbing this interaction with an unlabeled H3(1–12)K4me3-peptide which should ultimately lead to a decrease of the Alpha signal. Therefore, we prepared a serial dilution series of unlabeled H3(1–12)K4me3 in buffer spanning a range of 12 concentrations around the reported *K_D_* value of the H3K4me3-Spindlin1 interaction of 250 nM (determined by ITC)(15) and conducted the Alpha assay as described in the method section. In the same experiment we wanted to investigate the time-course of the binding between Spindlin1 and biotin-H3(1–23)K4me3 or unlabeled H3(1–12)K4me3, respectively. Therefore, we compared the results arising from experiments with four different incubation periods (15, 30, 60 and 90 min) after combination of His-Spindlin1, biotin-H3(1–23)K4me3 and unlabeled H3(1–12)K4me3. A mixture of Ni-NTA acceptor- and streptavidin donor beads was added to each well under subdued light (with respect to the light-sensitivity of the donor beads) and the plates were incubated for another 120 min. After readout of the microplate the recorded Alpha signals were used to calculate the inhibitory effect of unlabeled H3(1–12)K4me3 toward the interaction of biotin-H3(1–23)K4me3 and Spindlin1. IC_50_ values were calculated by fitting the inhibition values to a sigmoidal dose-response curve. Figure 1C shows the resulting dose response curve indicating a significant competition of unlabeled H3(1–12)K4me3 with biotin-H3(1–23)K4me3 for binding to His-Spindlin1 with IC_50_ values of 117.5 ± 3.6, 117.9 ± 3.3, 118.5 ± 1.7 and 115.5 ± 3.7 nM after the application of incubation times of 15, 30, 60 and 90 min, respectively.

As potential small-molecule ligands are usually dissolved in DMSO for inhibition experiments, we investigated the DMSO tolerance of our AlphaLISA assay in order to circumvent any effects on the assay signal not arising from potential competitors but from DMSO. We therefore prepared a serial dilution series of DMSO in assay buffer covering a concentration range of 0.125% to 10% in the final assay volume before adding the previously preincubated His-Spindlin1/biotin-H3K4me3 mixture and the Alpha bead mixture. Control wells contained buffer instead of DMSO and the resulting Alpha signals were used to normalize the values obtained from the DMSO containing samples. The results from this experiment show that DMSO concentrations up to 10% do neither influence the assay signal significantly nor is the Spindlin1/biotin-H3K4me3 interaction being disturbed (see Figure 1D). Optimization for this assay was completed by investigating the optimal incubation time after adding the Alpha beads to the assay mixture. Therefore, we analyzed incubation times of 10, 30, 60 and 120 min for this step. These tests were conducted on separate 384-well microplates onto which 32 positive and 32 negative controls were dispensed, respectively. The collected Alpha signals were also used to calculate the Z’-factor for each condition which served as an indicator of reproducibility and robustness of the assay. An incubation time after addition of the Alpha beads to the assay mixture exceeding 30 min was proven beneficial with regard to the overall assay window but no further improvement is gained by extending incubation times of the Alpha beads to more than 60 min (see Figure 1E). Bead incubation times of 30, 60, 90 and 120 min revealed Z’-Factors of 0.93, 0.89, 0.92 and 0.92, respectively. Taken together, all assay conditions provided results that match the requirements for a robust assay (19) (Z’-Factor > 0.5). To demonstrate the broader versatility of the Alpha assay for methyl lysine readers, we extended the application of the system to the double Tudor domain of the lysine demethylase JMJD2C (JMJD2C-Td). JMJD2C-Td is known to recognize the same histone modification (H3K4me3) as Spindlin1 (25). Thus, following the same optimization process as explained for Spindlin1 (data not shown), we were able to monitor binding between JMJD2C-TD and biotin-H3(1–23)K4me3 peptide and to show displacement of biotin-H3(1–23)K4me3 peptide from the JMJD2C-Td complex by unlabeled H3(1–12)K4me3 peptide (IC50 4.5 ± 0.2 μM, see Figure 1F and G).

### Fluorescence polarization assay

#### Principle and validation

**Figure 2.**
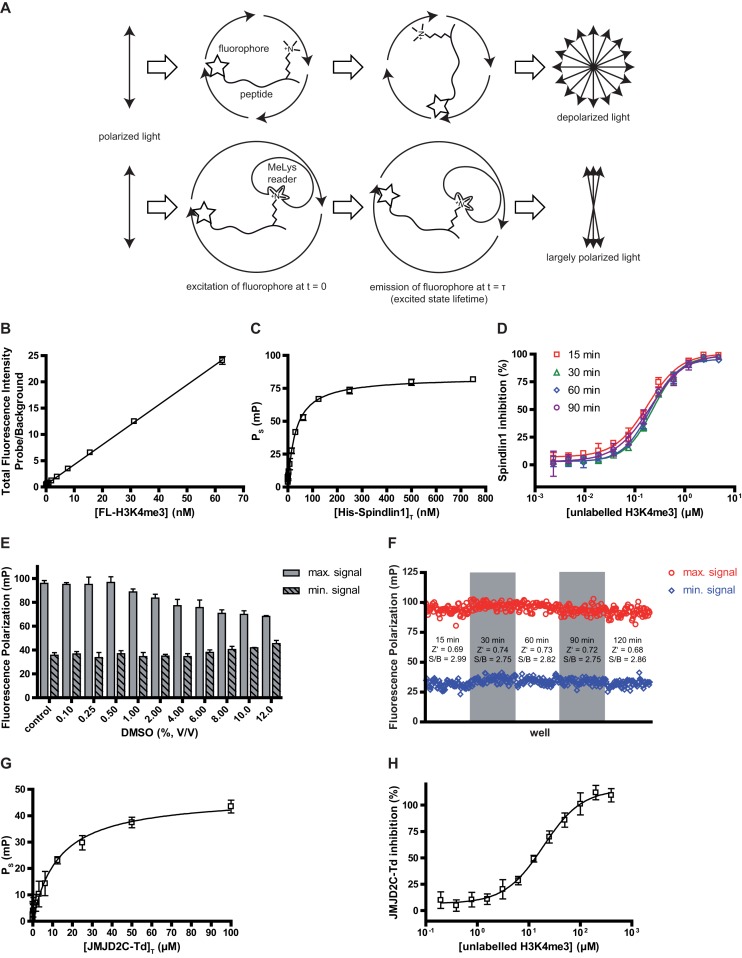
Setup of the fluorescence polarization (FP) based binding assay. (**A**) Principle of the FP-assay for the quantitation of reader proteins binding to a fluorescent probe. (**B**) Linearity of the total fluorescence intensity of FL-H3K4me3. Data of the lower concentration range (n = 15, measured range 0.06 to 750 nM) is presented as mean and standard deviation of triplicate measurements (linear fit: R^2^ = 0.9992). (**C**) *K_D_* determination of the interaction between FL-H3K4me3 and Spindlin1. Fluorescence polarization due to specific Spindlin1 binding (P_S_) was plotted against a panel of 15 total Spindlin1 concentrations (0.06–750 nM) and a one-site binding fit (hyperbola) was applied. Data represent the mean and standard deviation of triplicate measurements. (**D**) H3(1–12)K4me3 dose-response inhibition curve of Spindlin1. Unlabeled H3K4me3 peptide competed with 10 nM FL-H3K4me3 for binding to 100 nM His-Spindlin1. Assay mixtures were incubated for 15, 30, 60 or 90 min before fluorescence polarization measurements. Data represent the mean and standard deviation of triplicate measurements. (**E**) DMSO tolerance of the FP-assay. A panel of DMSO concentrations was exposed to the FP-assay mixture and the resulting influence on the maximum- and minimum signal was plotted. Data represent the mean and standard deviation of triplicate measurements. (**F**) Evaluation of incubation time and Z’-factors. Fluorescence polarization signals of 32 microplate wells containing positive and negative control assay mixtures were plotted after incubation periods of 15, 30, 60 90 and 120 min. (**G**) Transfer of the FP-assay setup to the double Tudor domain of JMJD2C. *K_D_* determination of the binding of FL-H3K4me3 to JMJD2C-Td. (**H**) Dose-response inhibition curve of unlabeled H3(1–12)K4me3 which competed with 15 nM FL-H3K4me3 for binding to 10 μM JMJD2C-Td. Data represent the mean and standard deviation of triplicate measurements.

If a sample containing a fluorescent moiety is excited with plane polarized light, it will emit light with a certain degree of polarization dependent on the size of the fluorescent molecule. As the degree of polarization of the released light is inversely proportional to the rate of molecular rotation, small fluorescent structures will largely depolarize the emitted light due to reorientation of the fluorophore during its excited state lifetime. In contrast, larger structures with attached fluorophores will retain the polarization of the excitation light to a greater extent (see Figure 2A). Fluorescence polarization (FP) is typically indicated as mP value which can be calculated after measuring the fluorescence intensities of the emitted light from a parallel (|$\parallel$) and from a perpendicular (|$\perp$) direction in relation to the excitation plane (26). This observation allows us to use FP as a technique for the measurement of ligand binding.

Thus, in a mixture of fluorescently labeled ligand and receptor the observed fluorescence polarization is a function of the fraction of bound ligand. For competitive ligand screening a decrease of the fraction of the receptor bound probe can be observed by a decrease of fluorescence polarization after co-incubation of the respective analyte with a preformed complex of receptor (i.e. a reader protein) and fluorescent probe.

#### Design of a fluorescent probe

We started the FP assay development with designing an appropriate fluorescent probe. Our goal was to set up an orthogonal assay for the previously established AlphaLISA assay. Again, we intended to target the aromatic cage of Spindlin1's Tudor-like domains. We thus decided to design a probe based on a peptide fragment derived from the N-terminal tail of histone protein H3 trimethylated at position 4 (H3K4me3). Naturally, the attachment of a fluorescent label to the ligand should ideally not affect its binding mode nor should it decrease the affinity of the ligand to the receptor (27). In previous studies it was shown that the amino acids 1–8 of histone H3 contribute most to the interaction between H3K4me3 and the second of the three Tudor-like domains of Spindlin1 (15). Thus, we concluded that a peptide sequence made up of amino acids 1–8 of H3K4me3 will display a suitable binding motif for Spindlin1. The amino acid sequence of the H3 tail further contains a GGK-sequence in position 12–14 which we chose to use as a linker for the addition of the fluorescent moiety fluorescein (FL-H3K4me3, see method section).

#### Determination of the K_D_ between the probe and Spindlin1

We initially performed a probe titration by serially diluting the probe in assay buffer. The total fluorescence intensities were calculated and the resulting values plotted against the respective probe concentrations. With this, linearity of the total fluorescence intensity of the probe could be shown up to a concentration of 750 nM (R^2^ = 0.9992). For 10 nM of the fluorescent probe significantly higher total fluorescence intensities could be observed compared to buffer (see Figure 2B). Furthermore, the calculation and plotting of mP values indicated a stable signal of about 25 mP at concentrations of the probe exceeding 5 nM (data not shown).

To obtain an appropriate assay window and a robust ligand screening assay, the fluorescent probe has to bind to the protein of interest (receptor) with high affinity. Thus, to evaluate the binding affinity (i.e. the *K_D_*) of FL-H3K4me3 to Spindlin1 we initially established binding curves by titrating a dilution series of Spindlin1 against the fluorescent probe. As the probe concentration should not surpass its *K_D_* with the receptor, to avoid stoichiometric titration (28), a probe concentration of 10 nM in the final assay volume was selected. This is also in accordance with our probe-titration results mentioned before. Next, a serial Spindlin1 dilution series was prepared and incubated with and without (for background correction) FL-H3K4me3 on a microplate. Fluorescence polarization was measured from these wells after an incubation step. Furthermore, all Spindlin1 dilutions were combined with FL-H3K4me3 together with a saturating concentration of an unlabeled H3K4me3-derived peptide. Thus, by simulating a situation where the probe is completely displaced by a competitive ligand, we were able to calculate the contribution of unspecific binding to the measured fluorescence polarization. The obtained values enabled us to calculate specific binding of FL-H3K4me3 to Spindlin1. From the obtained specific binding values we determined a *K_D_* of 31 nM for the interaction between FL-H3K4me3 and Spindlin1 which suggests that FL-H3K4me3 is a suitable high-affinity probe for our assay (see Figure 2C).

#### FP displacement assay

The interruption of the interaction between the fluorescent probe (D*) and Spindlin1 (R) by a competitive ligand of the Spindlin1 Tudor-like domain should lead to a decrease of the fluorescence polarization signal (P) as the probe is displaced from the complex (P_D*R_ → P_D*_). Therefore, to set up a suitable displacement assay for the screening of FL-H3K4me3 competitors, we had to choose appropriate concentrations of both, Spindlin1 and FL-H3K4me3. We maintained the FL-H3K4me3-concentration at 10 nM in the final assay volume. The Spindlin1 concentration should be on the one hand ideally set in a range, where saturated binding of the probe is ensured (see binding curve in Figure 2). On the other hand, for potential high-throughput screening target protein consumption should be as low as possible which demands a compromise between an appropriate assay window and an economic use of the target. In consideration of this, we set the Spindlin1 concentration in the assay to 100 nM. Displacement of the fluorescent probe from Spindlin1 was examined by performing an IC_50_ determination experiment as described in the method section, in which we used the unlabeled H3(1–12)K4me3 peptide as a reference competitor. We further used this experiment to examine the influence of incubation duration on the inhibition values and therefore dispensed the sample mixtures on four separate microplates and applied different incubation times to each plate (15, 30, 60 and 90 min). A dose-dependent displacement of the probe from Spindlin1 by the reference competitor could be clearly shown after plotting the inhibition values against the respective logarithmic ligand concentrations. Applying a sigmoidal dose-response fit provided inhibition curves for each incubation condition from which IC_50_ values of 180.5 ± 13.3, 231.8 ± 11.3, 201.6 ± 9.9 and 201.0 ± 17.0 nM were derived for incubation periods of 15, 30, 60 and 90 min respectively (see Figure 2D). These values are very much in accordance with the results from the previously described AlphaLISA assay. Furthermore, a quick formation of the binding equilibrium was indicated by the narrow range of IC_50_ values obtained after different incubation periods. Thus, the incubation time for subsequent experiments can be set in a relatively wide window.

We observed no disturbance of the assay signal by the ingredients of the AlphaLISA assay buffer. Thus, we decided to use the same buffer composition for both assay formats. The examination of DMSO influence on the assay signal and Z’-factor determinations were performed using the same strategies as described for the Alpha based assay. The results of the optimization steps are depicted in Figure 2E and F. At DMSO concentrations exceeding 1% in the final assay volume the assay window decreases markedly (S/B = 2.62, 2.57 and 2.39 for DMSO concentrations of 0.5%, 1% and 2% respectively). Hence, higher DMSO concentrations during the screening of DMSO-stock libraries should be avoided. An average Z’-factor of 0.7 indicates an excellent assay (Z’ > 0.5)(19). Again, readouts after 15, 30, 60, 90 and 120 min revealed consistent Z’ factors and signal-to-background relations. However, for subsequent experiments plates were incubated for 30 min prior to readouts in order to ensure consistent assay completions among different measurements.

We also checked the versatility of this assay for other targets by applying it to the double Tudor domain of JMJD2C (JMJD2C-Td). For this we made use of the same fluorescent probe as described for the Spindlin1 setup. Figure 2G and H summarizes the results obtained from the *K_D_*-determination- and H3K4me3-competition experiment. We determined a *K_D_* of 14.5 ± 1.9 μM for the FL-H3K4me3-JMJD2C interaction and an IC_50_ of 19.1 ± 2.1 μM for the unlabeled H3K4me3 peptide.

### *In vitro* fluorescent thermal shift assay

#### Principle and validation

We performed a FTSA, also referred to as differential scanning fluorimetry (DSF) or ThermoFluor^®^(20), to investigate stabilization effects of proteins that usually occur upon binding of a ligand. With the help of a hydrophobic, fluorescent dye (SYPRO Orange) whose fluorescence is increased upon binding to lipophilic protein regions and diminished from the quenching effect of water (29), the thermal unfolding of proteins can be monitored using a real-time PCR instrument. Ligand-induced stabilizing effects can be assessed from shifts of the unfolding temperature of the protein (T_m_) obtained in the presence of ligands relative to the T_m_ measured in the absence of ligands (30). The stability of a protein is related to its Gibbs free energy of unfolding (ΔG_u_) which is temperature-dependent (31). If the temperature is increased, ΔG_u_ normally decreases and becomes zero when the concentrations of folded and unfolded protein are equal. At this equilibrium, the temperature is termed as melting temperature (T_m_) of the protein. Upon ligand-binding to a protein the free energy contribution of this event in most cases results in an increase in ΔG_u_, which eventually may lead to an increase in T_m_ (20). Melting curves of the protein can be recorded by constantly measuring the fluorescence of SYPRO Orange as it is being attached to the protein's hydrophobic inner regions which are exposed during unfolding at increasing temperature. By fitting the fluorescence values in relation to the temperatures, the transition midpoints of the resulting melting curves can be calculated. Comparison of the temperature of this midpoint in the presence and absence of a ligand reveals T_m_-shifts that are related to the binding affinity of the ligand to the protein (20).

#### Spindlin1 fluorescent thermal shift assay

**Figure 3.**
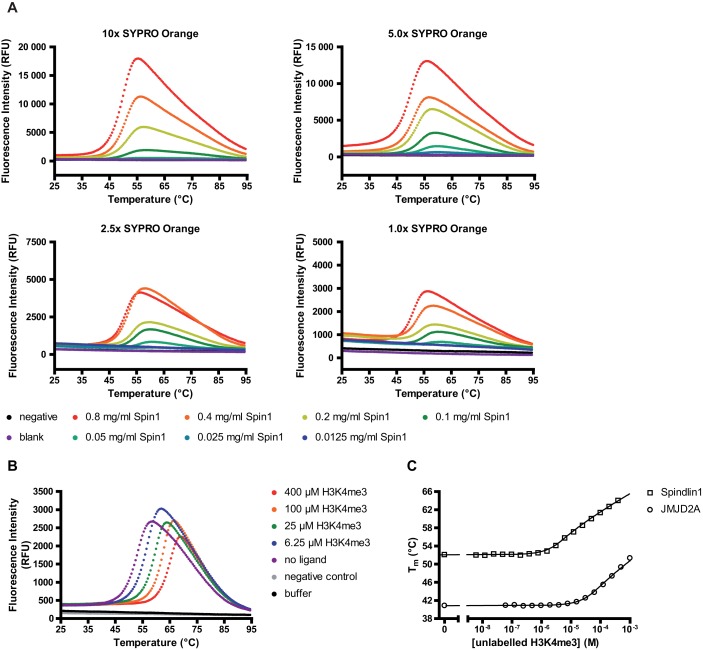
Setup of the FTSA for direct binding measurements. (**A**) Evaluation of appropriate His-Spindlin1 and SYPRO Orange concentrations for FTSA. Four concentrations of SYPRO Orange were titrated against seven concentrations of His-Spindlin1. Melting curves for all conditions were recorded by measuring the fluorescence intensity of SYPRO Orange during exposition to a temperature gradient (25–95°C, 0.5°C intervals). Negative controls contained only SYPRO Orange and blank controls contained buffer. Data of representative single runs are shown. (**B**) Melting curve shifts (ΔT_m_) of His-Spindlin1 upon ligand binding. Melting curves of native His-Spindlin1 or of His-Spindlin1-H3(1–12)K4me3 complexes after exposure to H3(1–12)K4me3 solutions of varying content. Stabilization of Spindlin1 upon H3(1–12)K4me3 binding resulted in ligand-dose-dependent shifts of the melting curves toward higher temperatures. Data of representative single runs are shown. (**C**) Plotting of T_m_ values against ligand concentration and KD simulation of the interaction between His-Spindlin1 and H3K4me-peptide allowed fitting and calculation of *K_D_* (according to (21–23)) for H3(1–12)K4me3-peptide interacting with His-Spindlin1 and JMJD2C-Td, respectively. Data represent the mean of triplicate measurements.

The optimal concentrations of the target protein Spindlin1 and the fluorescent dye SYPRO Orange needed to be evaluated, respectively. We conducted dilution series of SYPRO Orange and Spindlin1 which were cross-titrated on a PCR-plate. SYPRO Orange is provided as a 5000x stock solution in DMSO by the manufacturer without explicit reference to the actual concentration. Therefore, solutions of SYPRO Orange are indicated as dilutions based on this factor. Melting curves of each sample were recorded in a real-time PCR machine following the procedure described in the method section. A combination of 5x SYPRO Orange and 0.1 mg/ml Spindlin1 in solution was found to yield an optimal compromise between a well detectable melting curve and remote protein consumption (see Figure 3A). For a solution composed of 5x SYPRO Orange and 0.1 mg/ml Spindlin1, a melting temperature of 54.0°C was determined. A negative control containing only 5x SYPRO Orange diluted in buffer provided no increase in fluorescence over the complete span of the applied temperature gradient.

We chose to use a rather simple buffer composition for our experiments in order to be able to detect significant T_m_ shifts upon binding of ligands. An assay buffer composed of strongly stabilizing components would result in an already high T_m_ and might decrease the possibility to detect further stabilization by a potential ligand through T_m_-shifts toward higher temperatures. Thus, for subsequent experiments a buffer consisting of 100 mM Hepes (pH 7.5) and 150 mM NaCl was used. We noted that increasing NaCl concentration leads to drastic stabilization of Spindlin1. Other additives, like KCl or MgCl_2_, had no major impact on T_m_ of Spindlin1. Optimal buffer conditions were demonstrated for a neutral pH range (data not shown). It is further of note that detergents like Tween or CHAPS should be avoided in FTSA-assay buffers as they lead to drastic increase of the background fluorescence signal.

The possibility to identify new Spindlin1 binders with the help of this assay was evaluated by using the native ligand H3(1–12)K4me3 as a reference binder. We prepared a serial dilution series of H3(1–12)K4me3 peptide in assay buffer and performed the FTSA as described in the method section. Figure 3B illustrates the resulting dose-dependent T_m_ shifts of the protein upon ligand addition compared to the untreated protein after FTSA-readout. Co-incubation of Spindlin1 and increasing concentrations of competitive ligand led to significant dose-dependent shifts of the Spindlin1-melting curves towards higher temperatures compared to the native protein. From the T_m_ shifts, a *K_D_* value of 380 nM was calculated for the Spindlin1-H3K4me3 interaction (see Figure 3C).

Similar strategies as mentioned above have been used to establish FTSA for the exploration of JMJD2C double Tudor domain (JMJD2C-Td) ligands. In this case concentrations of 0.4 mg/ml JMJD2C-Td and 5x SYPRO Orange have been considered to be optimal during ligand characterizations. A *K_D_* value of 12.5 μM has been determined for the interaction between JMJD2C-Td and H3K4me3-peptide (see Figure 3C).

### BLI assay

#### Principle of the assay

**Figure 4.**
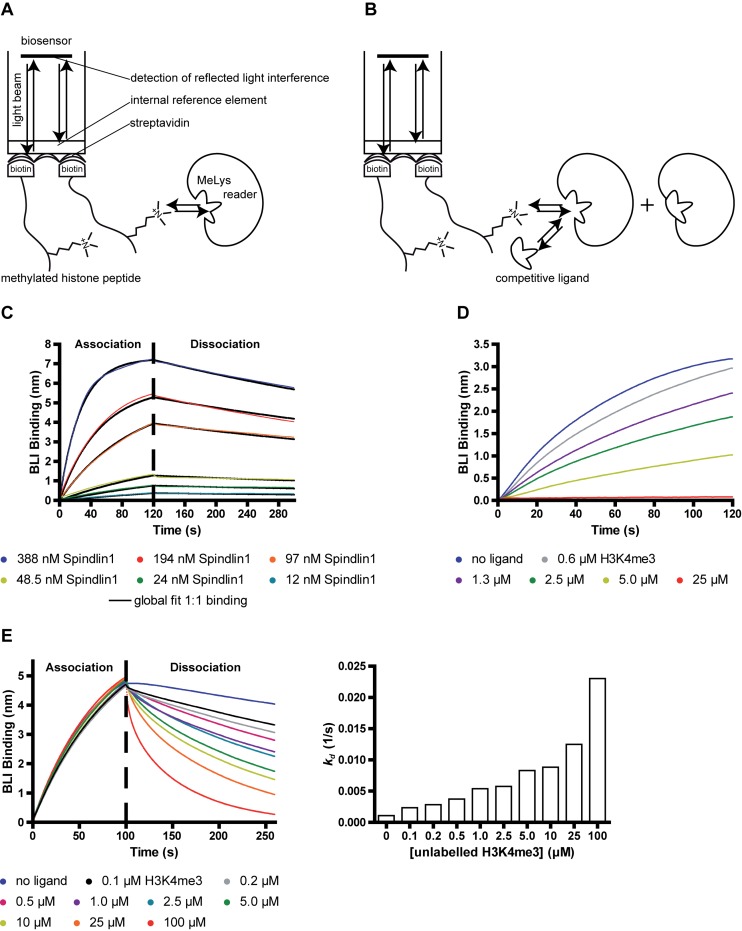
Setup of the BLI based assay. (**A**) Illustration of the assay-principle. Direct measurement of association and dissociation of reader protein to/from biotin-ligand immobilized on a SA-biosensor tip enables the determination of *K_D_* for this interaction. (**B**) Competitive setup of the assay: a fixed concentration of reader protein is preincubated with potential ligands competing with biotin-ligand immobilized on a SA-biosensor tip. (**C**) Association- and dissociation-sensorgrams of runs performed with various Spindlin1 concentrations using previously loaded biosensors. A buffer reference sensorgram was subtracted from all other curves. (**D**) Displacement setup of the BLI-assay. Dose-dependent decrease of the maximum signal of the association curve after preincubation of a fixed His-Spindlin1 concentration of 200 nM with increasing concentrations of unlabeled H3(1–12)K4me3. (**E**) Alternatively, the increase in *K_d_* is analyzed after immersing a biotin-H3(1–23)K4me3-loaded biosensor tip, to which His-Spindlin1 had been associated, into samples containing unlabeled H3(1–12)K4me3 in various concentrations. Data are representing single measurement runs.

Biolayer interferometry is a label-free technology suitable for measuring biomolecular interactions. The principle is based on the analysis of the interference pattern of white light reflected from two surfaces: a beam of white light which is sent through a biosensor made of a biocompatible matrix is reflected in two ways at the tip of the sensor, namely from a layer of immobilized ligand on the biosensor tip, and from an internal reference layer. In our setup we take advantage of streptavidin-coated biosensors which enable the immobilization of biotinylated ligands to the biosensor tips. Unlike other biosensor-based techniques, like for example SPR, BLI instruments do not require complex fluidic systems (32). Any change in the number of molecules bound to the biosensor tip, e.g. by dipping disposable biosensors with a ligand immobilized to the tip into an analyte solution, alters the optical thickness of the surface and thus causes a shift in the interference pattern (measured in nm) that can be plotted in real-time. This enables the user to record so-called sensorgrams of binding events including association and dissociation processes. Subsequently, association- and dissociation rates (*k_a_* and *k_d_*) or *K_D_* can be calculated by globally fitting the resulting sensorgrams to a 1:1 binding model. We set up a BLI-based binding assay (see Figure 4A) as well as a displacement assay (see Figure 4B) using the BLItz^®^ instrument.

#### Determination of the K_D_ value of the biotin-H3K4me3/Spindlin1 interaction via BLI

As the binding events are monitored by the change in optical thickness of the sensor tip surface, it is recommended to immobilize the smaller binding partner to the sensor tip and to keep the larger binding partner in the sample solution to achieve a significant change of optical thickness upon analyte binding to the tip. We thus decided to use the same biotinylated H3(1–23)K4me3 peptide that we also used for the AlphaLISA assay for immobilization to the biosensor tips. The maximum signal at the end of the loading step averaged 1.5 nm. It is advisable to optimize the loading step in terms of duration and ligand concentration. Extending the loading time indicated saturation of the binding capacity of the biosensor tips. This state should be avoided as a sensor tip saturated with immobilized ligand could potentially impede the binding of subsequently added binding partners by steric hindrance and thus reduced flexibility of the immobilized ligand. In a next step Spindlin1 solutions were prepared in six different concentrations and for each condition association- and dissociation curves were measured with the BLItz instrument following the procedures described in the method section. By applying a global 1:1 fit to all curves *k_a_, k_d_* and *K_D_* values were calculated (see Figure 4C). To determine a *k_d_* the binding responses should contain a minimum decrease of 5% in signal during the dissociation phase of the binding cycle (33). We obtained a *K_D_* value of 14.27 nM (with *k_a_* = 9.43 × 10^4^ (M·s)^−1^ and *k_d_* = 1.35 × 10^−3^ s^−1^) for the binding of Spindlin1 to the immobilized biotin-H3K4me3 peptide. Additionally, a biosensor was blocked with just biotin which is used to determine unspecific binding of the Spindlin1 samples. No significant unspecific binding of the examined Spindlin1 samples to the blocked biosensor was observed (data not shown).

#### Implementation of the BLI assay for competitive ligand screening

In order to be able to validate larger numbers of compounds for their ability to disrupt the Spindlin1-H3K4me3 interaction, we sought a way to set up a competitive assay format for the BLI assay (see Figure 4B). We pursued two different approaches. For the first strategy, a biotinylated H3K4me3 peptide was initially immobilized to the tip of a streptavidin functionalized biosensor and the potential competitor was preincubated together with a fixed concentration of Spindlin1 in a reaction tube. Another solution without competitor but with the same concentration of Spindlin1 was similarly prepared. Next, association- and dissociation-sensorgrams were recorded for both solutions. We then set report-points at the end of the association phase which allowed us to compare the respective BLI signal at the indicated time point. A decay of the signal of the curve containing Spindlin1 and unlabeled H3K4me3 was observed proving that the Tudor-like domain of Spindlin1 was blocked by unlabeled H3K4me3 and thus less binding of Spindlin1 to biotin-H3K4me3 on the biosensor tip occurred. We could also show a dose-dependent change of the BLI signal at the end of the association phase by measuring preincubated mixtures of a fixed Spindlin1 concentration with varying concentrations of competitor (see Figure 4D). In another approach, we investigated the effect of a competitive ligand on the *k_d_* of a preformed Spindlin1/biotin-H3K4me3-complex on the biosensor tips. Therefore, biotin-H3K4me3-loaded and Spindlin1-associated sensor tips were immersed into solutions containing a competitor and the recorded dissociation rate was compared to *k_d_* values obtained from using pure buffer instead of a competitor. Thus, it was also possible to detect an increased Spindlin1 displacement from the biosensor tips in the presence of a biotin-H3K4me3 competitor in a dose-dependent manner (see Figure 4E).

#### Regeneration of loaded SA-biosensors

**Figure 5.**
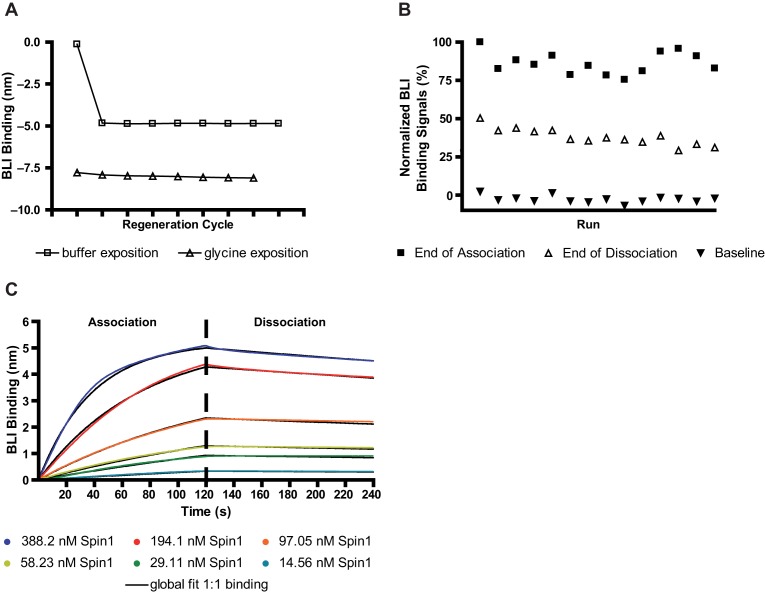
Regeneration of biotin-peptide-loaded SA-biosensors for the BLI-assay. (**A**) Plotting of BLI binding signals at the end of repeating washing steps. Alternating washing steps (20 s pure buffer and 10 mM glycine, pH 1.5, respectively) were applied to a SA-biosensor that has been exposed to 400 nM Spindlin1 for binding. A large decay of the binding signal after the first washing step indicated efficient removal of residual Spindlin1 from the biosensor tip. The baseline signal stayed stable after the second washing step. (**B**) Use of regenerated BLI biosensors. Stability of the assay window was proven for up to 15 repetitive regeneration cycles of the same loaded biosensor tip. (**C**) *K_D_* determination experiment using a regenerated biosensor tip for association and dissociation measurements of six different Spindlin1 concentrations (starting with the lowest protein concentration). Data are representing single measurement runs.

As we were planning to test and characterize larger numbers of potential inhibitors of the Spindlin1-H3K4me3 interaction we established a regeneration protocol for loaded biosensor tips in order to minimize the consumption of expensive biosensors and biotin-H3K4me3 solution. Residual amounts of Spindlin1 were washed off the biosensor tip after the dissociation measurements by applying alternating washing steps in pure BLI buffer and in 10 mM glycine solution (pH 1.5). Report points were set at the end of each step to evaluate the efficiency of the washing procedure and the stability of the buffer baseline (see Figure 5A). A washing procedure including three cycles of alternating exposure to buffer and to 10 mM glycine (pH 1.5) was adequate and was hence adopted to our assay protocol. Using this washing procedure, association signals obtained from one regenerated biosensor stayed stable over up to 15 runs (see Figure 5B). Repeating the *K_D_* determination for the interaction between Spindlin1 and biotin-H3K4me3 with one biosensor which was regenerated after each run revealed a *K_D_* value of 13.2 nM (with *k_a_* = 6.54 × 10^4^ (M·s)^−1^ and *k_d_* = 8.63 × 10^−4^ s^−1^) which is in the same range as the *K_D_* value obtained from the experiment using fresh biosensors for every run (see Figure 5C).

### CETSA

#### Principle and validation

We intended to use the so-far described *in vitro* assays for identification and verification of new small-molecule ligands of Spindlin1 in screening campaigns. We also wanted to be able to further characterize the binding properties of compounds in a cellular context. Thus, we planned to establish a simple assay-setup that would enable us to conveniently monitor the binding of a potential ligand to Spindlin1 in a native cellular background. We therefore set up a CETSA for the investigation of cell penetration and subsequent target engagement of potential small-molecule ligands of Spindlin1 (14). Practically, the most important steps of CETSA involve treatment of cells with potential small-molecule ligands of Spindlin1, heating of the cell suspension to denature and thus precipitate proteins, cell lysis by repeating freeze-thaw cycles and the separation of cell debris and precipitated protein aggregates from the soluble protein fraction. CETSA is based on a similar principle as already used for the *in vitro* thermal shift assay on recombinant Spindlin1. At increased temperatures native proteins tend to unfold and aggregate whereas a thermodynamic stabilization effect is observed for ligand-bound proteins, keeping them in solution. Here, the stabilizing effect on Spindlin1 upon ligand binding is detected by denaturing gel electrophoresis (SDS-PAGE) and quantitative Western-blot analysis of the soluble fraction of cell lysates from cells that were treated with potential small-molecule ligands and exposed to a panel of different temperatures. Using cell lysates instead of whole cell suspensions from the start of the experiment offers a variant of the assay that allows to explore the binding of potential ligands to the intended target Spindlin1 without the need of cell penetration of the ligand. This setup would be beneficial for the characterization of larger, cell-impermeable ligands, like for example peptides, in a more physiological context as compared to an *in vitro* assay.

#### Setting up CETSA for Spindlin1

**Figure 6.**
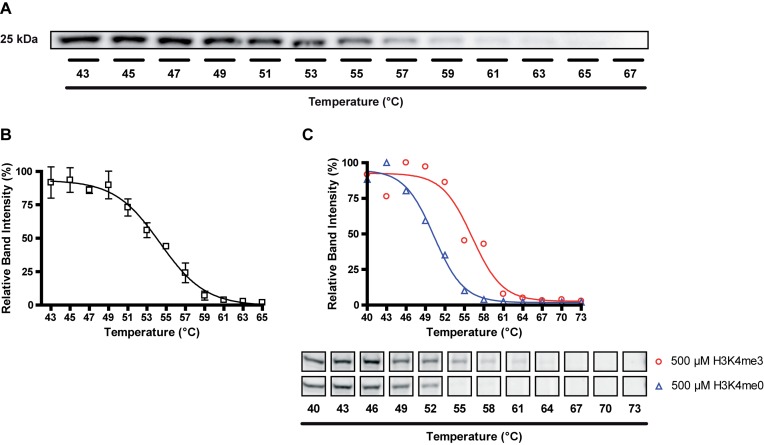
CETSA setup for Spindlin1. (**A**) Representative Western-blot signals corresponding to Spindlin1 show a decrease in intensity at elevated temperatures. (**B**) CETSA-melting curve of Spindlin1. Band intensities obtained from Western-blot analysis were related to the highest Western-blot signal which has been set to 100%. Relative Spindlin1-band intensities were plotted against corresponding incubation temperatures and a Boltzmann sigmoidal fit was applied. Data represent the mean and standard deviation of two individual experiments. (**C**) Spindlin1 CETSA curve shift upon ligand binding. Comparison of H3(1–12)K4me3- and H3(1–21)Kme0-treated (500 μM) HL-60 cell lysate. Underlying Western-blots are shown for clarification.

We were able to show that Spindlin1, expressed by HL-60 suspension cells, is adequately detected from the soluble fraction of the cell lysate by SDS-PAGE and Western-blot analysis with Spindlin1-specific antibodies (see Figure 6A). We thus chose HL-60 cells for setting up our CETSA assay. Samples containing equal amounts of HL-60 cells were each treated with a defined temperature during the heating step. As a proof-of-concept experiment we wanted to produce a melting curve of Spindlin1 in the absence of any stabilizing ligand. Thus, the apparent aggregation temperature (T_agg_) of Spindlin1 can be determined. During the curse of this experiment, previously reported general recommendations for CETSA addressing heating duration, cell lysis techniques and the separation of insoluble protein aggregates and cell debris from the remaining soluble protein fraction have been confirmed (14). The heating step was performed with a PCR-thermal cycler that allows the application of a temperature gradient of up to 16 temperatures in one heating block in parallel. For our protocol, we selected a heating duration of 3 min at 12 different temperatures covering a range between 40 and 73°C in 3°C intervals in parallel followed by a uniform cooling step for all wells at 25°C for another 3 min before the cell suspensions were shock-frozen in liquid nitrogen. It is of note that a homogeneous treatment of all samples throughout the whole process must be ensured in order to obtain reproducible and comparable results. This requires a precise thermal cycler that allows rapid heating and cooling to the desired temperatures within seconds with a minimum of temperature deviation during the heating and cooling steps. Alternatively, the samples could be inserted into the thermal cycler after the respective temperatures have been reached in order to avoid unwanted differences in the treatment of the samples by, e.g. inconsistent heating rates of the machine. Cell lysis was conducted by alternating thaw-freeze cycles at 25°C and in liquid nitrogen. Again, it is important to maintain a uniform treatment of the samples. Therefore, it is recommended to use a preheated thermoblock at 25°C for the thawing steps. Ultimately after cell lysis, the separation step of the soluble fraction from cell debris and aggregated proteins was conducted by centrifugation of the lysed cell suspension at 4°C. The supernatants of the samples were then submitted to SDS-PAGE and quantitative Western-blot analysis. The melting curve resulting from this experiment indicated an apparent T_agg_ of Spindlin1 of 54.4°C (see Figure 6B).

#### T_agg_ shifts of Spindlin1 in cell lysates

As no small molecule ligands had been described so far, our model ligand for Spindlin1, H3(1–12)K4me3, was chosen as a non-cell permeable stabilizer. Thus, we modified the CETSA setup to be able to evaluate the possibility of observing stabilizing effects on Spindlin1 by a bound ligand. We exposed a sample of previously lysed HL-60 cells to a fixed concentration of H3K4me3 peptide. Thus, cellular penetration processes do not impede the accessibility of the peptide ligand to Spindlin1. Another sample of lysed HL-60 cells that was treated with the unmethylated variant of the model ligand (H3(1–21)K4me0) was used as a control experiment. Following the ligand incubation step, the samples were aliquoted and heated as mentioned before. Comparison of the melting curves obtained from the ligand treated cell lysate with the curves resulting from the control experiment showed a significant shift of Spindlin1-T_agg_ toward higher temperatures (ΔT_agg_ = 5.9°C). This indicates stabilization of Spindlin1 upon binding of H3(1–12)K4me3 in the presence of cellular components (see Figure 6C).

### Application of the platform in small-molecule screening

**Figure 7.**
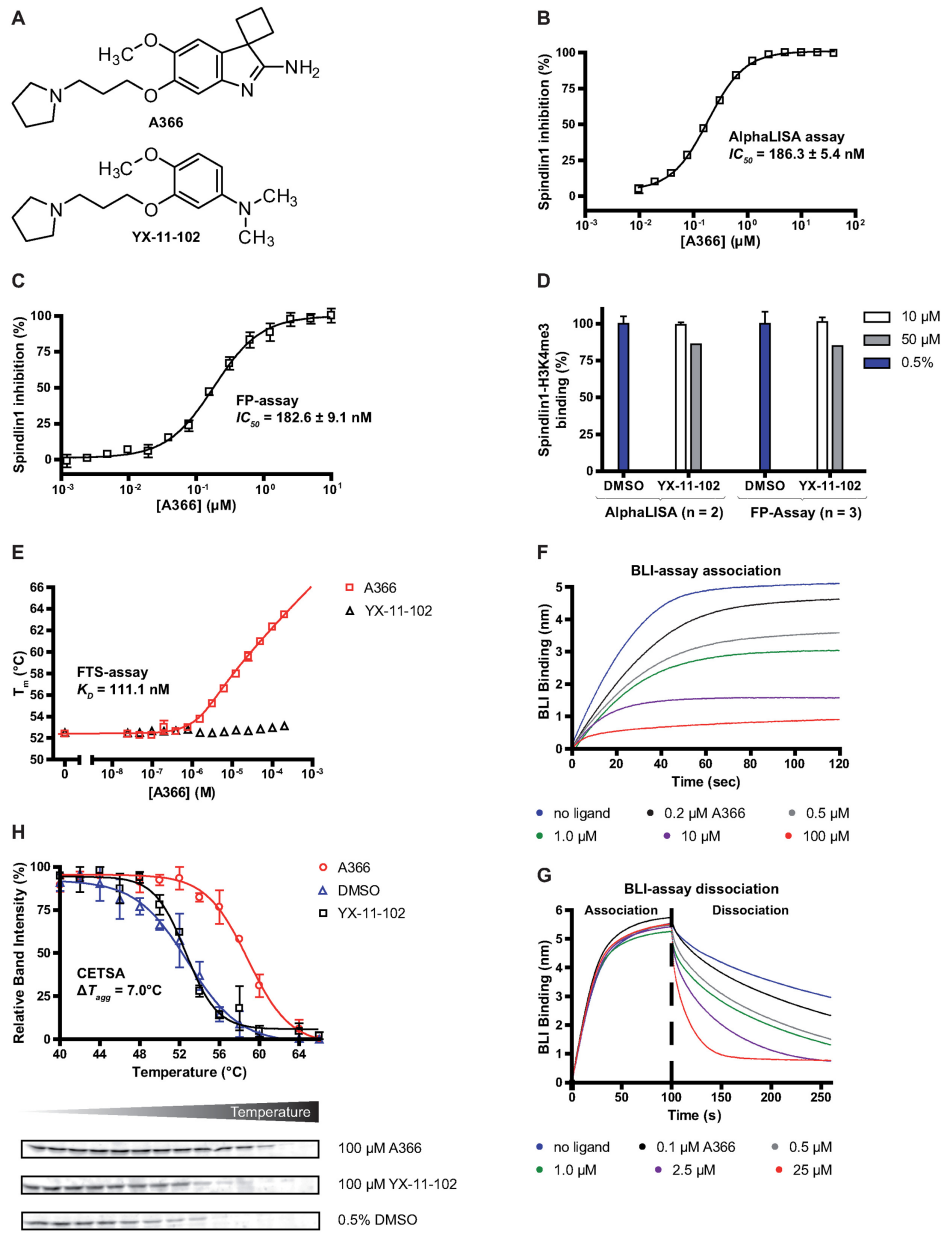
Identification and validation of A366 as potent small-molecule inhibitor of the Spindlin1-H3K4me3-interaction. (**A**) Structure of the Spindlin1 ligand screening hit A366 and the negative control compound YX-11–102. (**B**) A366 dose-response inhibition curve of Spindlin1 in the AlphaLISA assay. A366 competed with 30 nM biotin-H3(1–23)K4me3 for binding to 15 nM His-Spindlin1. Data represent the mean and standard deviation of triplicate measurements. (**C**) A366 dose-response inhibition curve of Spindlin1 in the FP-assay. A366 competed with 10 nM FL-H3K4me3 for binding to 100 nM His-Spindlin1. Data represent the mean and standard deviation of triplicate measurements. (**D**) AlphaLISA- and FP-assay screening results of YX-11–102 in comparison with DMSO. (**E**) Plotting of T_m_ values from FTSA against A366 concentration allowed fitting and calculation of *K_D_* (according to (21–23) for A366 interacting with His-Spindlin1. *T_m_* values obtained from FTSA with YX-11–102 are shown for comparison. Data represent the mean of triplicate measurements. (F) and (G) Displacement of His-Spindlin1 from biotin-H3(1–23)K4me3-loaded biosensors by A366 in the BLI-assay. Data represent single-run measurements. (**F**) Dose-dependent decrease of the maximum signal of the association curve after preincubation of a fixed His-Spindlin1 concentration with increasing concentrations of A366. (**G**) Increase in *k_d_* after immersing a biotin-H3(1–23)K4me3-loaded biosensor tip, to which His-Spindlin1 had been associated, into samples containing A366 in various concentrations. (**H**) CETSA indicated HL-60-cell penetration and binding of A366 to Spindlin1. HL-60 cells had been treated with A366, YX-11–102 or DMSO. Data represent the mean and standard deviation of two individual Western-blots. Representative Western-blots are shown for clarification.

After establishing and validating our assay platform we tested the screening applicability of our proposed workflow (see Scheme 1) by using our system for screening of a collection of commercially available bioactive compounds, including known epigenetic probes and selected inhibitors from in-house repositories as potential inhibitors of the Spindlin1-H3K4me3-interaction. Although the druggability of Tudor domains has been predicted to be rather challenging (34) we successfully identified A366 (35) (see Figure 7A) as a promising and potent small-molecule ligand of Spindlin1 which revealed an *IC_50_* of 186.3 ± 5.4 nM in the AlphaLISA assay (see Figure 7B) whilst showing no significant Alpha-interference in the TruHits-kit. This initial hit was confirmed using the FP-assay which accordingly showed an IC_50_ of 182.6 ± 9.1 nM (see Figure 7C). We furthermore found that the structurally related compound YX-11–102 did not show significant Spindlin1 binding in both AlphaLISA- and FP-Assays (see Figure 7D). We thus planned to further validate A366 as a Spindlin1 ligand and to use YX-11–102 as a negative control compound. Direct binding of A366 to the Tudor domain of Spindlin1 was supported by showing dose-dependent shifts of the melting curve of Spindlin1 preincubated with different amounts of A366 in our FTSA. Curve fitting and calculation of binding affinities resulted in determination of a *K_D_* 111.1 nM. No changes in *T_m_* values were observed in FTSA with the negative control compound YX-11–102 (see Figure 7E). A366 competes for binding to Spindlin1's Tudor domain with the native ligand H3K4me3 which was demonstrated with our BLI-assay. Overall association of Spindlin1 to biotin-H3K4me3-loaded biosensors was reduced after preincubation with A366 (see Figure 7F) and the rate of Spindlin1 dissociating from biotin-H3K4me3-loaded biosensors (*k_d_*) was increased in the presence of A366 (see Figure 7G). CETSA was performed with cultured HL-60 cells which have been incubated with 100 μM A366 or YX-11–102 and with DMSO as control. The presence of A366 led to a significant shift of *T_agg_* as compared to the DMSO control experiment (Δ*T_agg_* = 7.0°C) indicating cell penetration and Spindlin1 stabilization via specific binding of A366 to the Tudor domain. The negative control compound YX-11–102 did not significantly change *T_agg_* of Spindlin1 in comparison to DMSO (Δ*T_agg_* = 0.8°C, see Figure 7H). This underlined a specific effect of A366 on Spindin1, although relatively high concentrations of the respective compounds were used in CETSA experiments.

## DISCUSSION

Epigenetic reader proteins are receiving more and more attention in the field of drug discovery as increasing evidence is gained about their importance in the formation and progression of various diseases including cancers. Especially for acetyl lysine readers (bromodomain proteins) promising findings have been made and initial drug candidates are already in clinical studies (36). Until today, methyl lysine readers have been pursued as drug targets to a much lesser extent, e.g. because their druggability was estimated to be rather limited (34). However, a growing number of methyl lysine readers, including the main target of our study, Spindlin1 (18), have been identified as promising drug targets bringing up the demand for reliable and robust test systems enabling the screening for druglike ligands of methyl lysine readers. Such ligands bear a great potential for drug development and will be useful chemical probes for cellular studies. A limited number of small-molecules that block methyl lysine reader proteins have been reported ((9,13) and others reviewed in (2)), but however, for many potential targets, including Spindlin1, no small-molecule ligands are known yet.

We were able to develop a powerful, flexible and versatile assay platform that can aid in identifying new ligands of epigenetic methyl lysine reader proteins. Within our panel of different assay techniques we suggest to use the AlphaLISA assay as a primary screening tool of larger compound libraries as this method works very sensitively and reveals a large and reproducible assay window whilst consuming only small amounts of protein and peptide. However, the Alpha technology is somewhat prone to assay interferences potentially arising from fluorescence quenchers, biotin- or His-tag mimetics or light scatterers which might lead to false positive or false negative hits. Therefore, hits obtained from Alpha based screening approaches generally need to be further verified and validated. Some of these issues can be addressed with the help of the commercially available TruHits Kit (see Materials and Method section) and by additional characterization with orthogonal assay systems. Our FP displacement assay setup was shown to serve as an adequate orthogonal test system for the screening of competitive ligands of Spindlin1 in addition to the AlphaLISA assay and to confirm initial hits obtained from primary screenings. Both, the AlphaLISA assay and the FP assay are adaptable to medium- or high-throughput screening as they require very low amounts of consumables and they can be performed in small assay volumes on 384-well plates within a short time period.

The versatility of our screening platform is emphasized by its ability to be easily transferred to other reader domain containing proteins. For example, in this study we successfully adapted the AlphaLISA- and FP assay to the analysis of the double Tudor domain of the histone demethylase JMJD2C.

We also aimed to provide our platform with biophysical techniques to be able to detect direct binding between reader proteins and ligands. Therefore, we established that a FTSA is well applicable for further characterization of Spindlin1 ligands. Through its label-free principle, FTSA does not require any modifications of the reader protein or the ligand by means of fluorescent- or affinity marks. Ligand affinity to Spindlin1 can not only be quickly estimated by ranking of T_m_ obtained at a certain ligand concentration, but also binding constants can be determined. This makes FTSA a valuable alternative to methods like, e.g. ITC where much higher amounts of both reader protein and ligand are consumed. Furthermore, FTSA can be conveniently used to rapidly screen for stabilizing effects of buffer components and thus can help to optimize conditions for other experiments like for example protein crystallization efforts (37). Like the AlphaLISA- and FP assays, FTSA was also successfully transferred to the examination of the double Tudor domain of JMJD2C. Although the affinity of H3K4me3 peptide to JMJD2C-Td is weaker compared to Spindlin1, a *K_D_* (13.3 μM) could still be determined. To our knowledge, we present here the first thermodynamic binding data on the H3K4me3-JMJD2C-Td interaction.

Another technique to characterize the influence of competitive reader protein ligands on the kinetic parameters of a reader protein-probe interaction is presented by our novel BLI assay setup which is a well suitable method for the validation of ligand screening hits. Based on its biophysical principle, BLI is not prone to interferences arising from fluorescent properties of potential competitors, such as auto-fluorescence or quenching effects. To our knowledge, this is the first published displacement assay setup using the BLItz instrument that enables quick estimations of protein–protein-inhibitor potencies by evaluating their ability to disturb a preformed probe-protein complex.

Finally, the cellular thermal shift assay ideally complements the panel of previously introduced *in vitro* methods that use isolated, recombinant Spindlin1. Using HL-60 promyelocytic leukemia cells, CETSA reveals well-defined melting curves for Spindlin1 and can thus be employed to investigate whether an initially found ligand of recombinant Spindlin1 actually engages Spindlin1 also in a biological relevant setting. For peptide derived or non-cell-penetrating ligands the assay can be employed using cell lysates instead of intact cells onward from the ligand incubation step. For the CETSA we needed relatively high concentrations of the peptide to obtain significant stabilization. But this is no surprise and can easily be explained by a possible binding engagement of H3(1–12)K4me3 to several other cellular interaction partners potentially reducing the effective concentration of H3(1–12)K4me3 being exposed to Spindlin1. For example, H3(1–12)K4me3 might be trapped by binding to other reader proteins, DNA-fragments or further soluble cell components. Furthermore, H3(1–12)K4me3 might be modified by writer or eraser enzymes (e.g. histone demethylases) in a way that it cannot be adequately recognized by Spindlin1 anymore. But in absence of available small molecule ligands of Spindlin1 during the validation process of our platform, the peptide served as helpful positive control to show that Spindlin1 reacts to binding of ligands in the physiological setting by a strong stabilization. This is especially valuable for the analysis of target engagement of reader proteins in a native background.

The application of our platform was finally validated by screening a collection of potential small-molecule ligands of the Tudor domain of Spindlin1. Thus, we were able to identify and validate A366 as a potent, nanomolar inhibitor of the Spindlin1-H3K4me3-interaction which also showed cellular target engagement. Up to now only one small-molecule ligand of a Tudor-domain-containing protein has been reported. Perfetti *et al*. identified UNC2170 as a micromolar (*K_D_* = 22 ± 2.5 μM) fragment-like ligand of 53BP1, a reader of H3K36me2 (38) and H4K20me2 (39) marks (40). As A366 was initially published as inhibitor of the lysine methyltransferase G9a by the SGC (35), the establishment of selectivity for Spindlin1 towards G9a is subject of ongoing medicinal chemistry efforts in our group. In general, hits from screening that are potent and selective already from the first screen are very rare in drug discovery. Given the very good potency of A366 on Spindlin1, this is thus an excellent starting points toward potent and selective inhibitors of Spindlin1.

In our screening approach we also found YX-11–102 as a fragment-like analogue of A366 which showed no inhibition of the Spindlin1-H3K4me3 interaction in the AlphaLISA- and the FP-assay. Therefore, we chose YX-11–102 as negative control compound in our experiments to emphasize the specific binding of the A366 effects toward Spindlin1. Especially in a cellular context like in CETSA-experiments a valid negative control represents a useful tool to indicate target specificity of a hit compound. We think that A366, and eventually optimized derivatives thereof, together with YX-11–102 can serve as useful tool compounds to further explore the biological role of Spindlin1 in phenotypic studies.

In conclusion, our assay platform enables screening of small-molecule libraries and identification, verification and validation of hit compounds including cellular target engagement, and will help to provide novel potent and selective ligands for methyl lysine reader proteins. The assays described herein will aid to improve initially found lead structures for Spindlin1 or other methyl lysine readers by iterative rounds of medicinal chemistry and biological testing. Thus, we provide a whole comprehensive set of tools to establish high-quality chemical probes to explore the rich biology of methyl lysine readers and for the development of drugs targeting these methyl lysine readers. With A366, we discovered an unprecedented hit for Spindlin1 that is amenable for optimization and can be developed into promising chemical probes and potentially, drug candidates for further development.

## Supplementary Material

SUPPLEMENTARY DATA

## References

[1] Kouzarides T. (2007). Chromatin modifications and their function. Cell.

[2] Wagner T., Robaa D., Sippl W., Jung M. (2014). Mind the methyl: methyllysine binding proteins in epigenetic regulation. ChemMedChem.

[3] Taverna S.D., Li H., Ruthenburg A.J., Allis C.D., Patel D.J. (2007). How chromatin-binding modules interpret histone modifications: lessons from professional pocket pickers. Nat. Struct. Mol. Biol..

[4] Lu Q., Quinn A.M., Patel M.P., Semus S.F., Graves A.P., Bandyopadhyay D., Pope A.J., Thrall S.H. (2012). Perspectives on the discovery of small-molecule modulators for epigenetic processes. J. Biomol. Screen..

[5] Jenuwein T., Allis C.D. (2001). Translating the histone code. Science.

[6] Collins R.E., Northrop J.P., Horton J.R., Lee D.Y., Zhang X., Stallcup M.R., Cheng X. (2008). The ankyrin repeats of G9a and GLP histone methyltransferases are mono- and dimethyllysine binding modules. Nat. Struct. Mol. Biol..

[7] Kuo A.J., Song J., Cheung P., Ishibe-Murakami S., Yamazoe S., Chen J.K., Patel D.J., Gozani O. (2012). The BAH domain of ORC1 links H4K20me2 to DNA replication licensing and Meier–Gorlin syndrome. Nature.

[8] Frye S.V. (2015). Unlocking the potential of chemical probes for methyl-lysine reader proteins. Future Med. Chem..

[9] Herold J.M., Wigle T.J., Norris J.L., Lam R., Korboukh V.K., Gao C., Ingerman L.A., Kireev D.B., Senisterra G., Vedadi M. (2011). Small-molecule ligands of methyl-lysine binding proteins. J. Med. Chem..

[10] Ren C., Morohashi K., Plotnikov A.N., Jakoncic J., Smith S.G., Li J., Zeng L., Rodriguez Y., Stojanoff V., Walsh M. (2015). Small-molecule modulators of methyl-lysine binding for the CBX7 chromodomain. Chem. Biol..

[11] Quinn A.M., Bedford M.T., Espejo A., Spannhoff A., Austin C.P., Oppermann U., Simeonov A. (2010). A homogeneous method for investigation of methylation-dependent protein–protein interactions in epigenetics. Nucleic Acids Res..

[12] Karatas H., Townsend E.C., Bernard D., Dou Y., Wang S. (2010). Analysis of the binding of mixed lineage leukemia 1 (MLL1) and histone 3 peptides to WD repeat domain 5 (WDR5) for the design of inhibitors of the MLL1-WDR5 interaction. J. Med. Chem..

[13] James L.I., Barsyte-Lovejoy D., Zhong N., Krichevsky L., Korboukh V.K., Herold J.M., MacNevin C.J., Norris J.L., Sagum C.A., Tempel W. (2013). Discovery of a chemical probe for the L3MBTL3 methyllysine reader domain. Nat. Chem. Biol..

[14] Jafari R., Almqvist H., Axelsson H., Ignatushchenko M., Lundbäck T., Nordlund P., Molina D.M. (2014). The cellular thermal shift assay for evaluating drug target interactions in cells. Nat. Protoc..

[15] Yang N., Wang W., Wang Y., Wang M., Zhao Q., Rao Z., Zhu B., Xu R.-M. (2012). Distinct mode of methylated lysine-4 of histone H3 recognition by tandem tudor-like domains of Spindlin1. Proc. Natl. Acad. Sci. U.S.A..

[16] Wang J.-X., Zeng Q., Chen L., Du J.-C., Yan X.-L., Yuan H.-F., Zhai C., Zhou J.-N., Jia Y.-L., Yue W. (2012). SPINDLIN1 promotes cancer cell proliferation through activation of WNT/TCF-4 signaling. Mol. Cancer Res..

[17] Zhang P., Cong B., Yuan H., Chen L., Lv Y., Bai C., Nan X., Shi S., Yue W., Pei X. (2008). Overexpression of spindlin1 induces metaphase arrest and chromosomal instability. J. Cell. Physiol..

[18] Franz H., Greschik H., Willmann D., Ozretic L., Jilg C.A., Wardelmann E., Jung M., Buettner R., Schule R. (2015). The histone code reader SPIN1 controls RET signaling in liposarcoma. Oncotarget.

[19] Zhang J.H., Chung T.D., Oldenburg K.R. (1999). A simple statistical parameter for use in evaluation and validation of high throughput screening assays. J. Biomol. Screen..

[20] Niesen F.H., Berglund H., Vedadi M. (2007). The use of differential scanning fluorimetry to detect ligand interactions that promote protein stability. Nat. Protoc..

[21] Matulis D., Kranz J.K., Salemme F.R., Todd M.J. (2005). Thermodynamic stability of carbonic anhydrase: measurements of binding affinity and stoichiometry using thermofluor. Biochemistry.

[22] Cimmperman P., Baranauskienė L., Jachimovičiūtė S., Jachno J., Torresan J., Michailovienė V., Matulienė J., Sereikaitė J., Bumelis V., Matulis D. (2008). A quantitative model of thermal stabilization and destabilization of proteins by ligands. Biophys. J..

[23] Baranauskienė L., Hilvo M., Matulienė J., Golovenko D., Manakova E., Dudutienė V., Michailovienė V., Torresan J., Jachno J., Parkkila S. (2010). Inhibition and binding studies of carbonic anhydrase isozymes I, II and IX with benzimidazo[1, 2-c][1, 2, 3]thiadiazole-7-sulphonamides. J. Enzyme Inhib. Med. Chem..

[24] Bielefeld-Sevigny M. (2009). AlphaLISA immunoassay platform- the ‘no-wash’ high-throughput alternative to ELISA. Assay Drug Dev. Technol..

[25] Pedersen M.T., Agger K., Laugesen A., Johansen J.V., Cloos P.A.C., Christensen J., Helin K. (2014). The demethylase JMJD2C localizes to H3K4me3-positive transcription start sites and is dispensable for embryonic development. Mol. Cell. Biol..

[26] Lakowicz J.R. (2006). Principles of fluorescence spectroscopy.

[27] Huang X., Aulabaugh A. (2009). Application of fluorescence polarization in HTS assays. Methods Mol. Biol..

[28] Rossi A.M., Taylor C.W. (2011). Analysis of protein-ligand interactions by fluorescence polarization. Nat. Protoc..

[29] Matulis D., Baumann C.G., Bloomfield V.A., Lovrien R.E. (1999). 1-Anilino-8-naphthalene sulfonate as a protein conformational tightening agent. Biopolymers.

[30] Lo M.-C., Aulabaugh A., Jin G., Cowling R., Bard J., Malamas M., Ellestad G. (2004). Evaluation of fluorescence-based thermal shift assays for hit identification in drug discovery. Anal. Biochem..

[31] Schellman J.A. (1997). Temperature, stability, and the hydrophobic interaction. Biophys. J..

[32] Rouet R., Lowe D., Dudgeon K., Roome B., Schofield P., Langley D., Andrews J., Whitfeld P., Jermutus L., Christ D. (2012). Expression of high-affinity human antibody fragments in bacteria. Nat. Protoc..

[33] Katsamba P.S., Navratilova I., Calderon-Cacia M., Fan L., Thornton K., Zhu M., Bos T.V., Forte C., Friend D., Laird-Offringa I. (2006). Kinetic analysis of a high-affinity antibody/antigen interaction performed by multiple Biacore users. Anal. Biochem..

[34] Santiago C., Nguyen K., Schapira M. (2011). Druggability of methyl-lysine binding sites. J. Comput. Aided Mol. Des..

[35] Sweis R.F., Pliushchev M., Brown P.J., Guo J., Li F., Maag D., Petros A.M., Soni N.B., Tse C., Vedadi M. (2014). discovery and development of potent and selective inhibitors of histone methyltransferase G9a. ACS Med. Chem. Lett..

[36] Filippakopoulos P., Qi J., Picaud S., Shen Y., Smith W.B., Fedorov O., Morse E.M., Keates T., Hickman T.T., Felletar I. (2010). Selective inhibition of BET bromodomains. Nature.

[37] Cimmperman P., Matulis D. (2011). Biophysical approaches determining ligand binding to biomolecular targets: detection, measurement and modelling.

[38] Tong Q., Cui G., Botuyan M.V., Rothbart S.B., Hayashi R., Musselman C.A., Singh N., Appella E., Strahl B.D., Mer G. (2015). Structural plasticity of methyllysine recognition by the tandem tudor domain of 53BP1. Structure.

[39] Botuyan M.V., Lee J., Ward I.M., Kim J.E., Thompson J.R., Chen J., Mer G. (2006). Structural basis for the methylation state-specific recognition of histone H4-K20 by 53BP1 and Crb2 in DNA repair. Cell.

[40] Perfetti M.T., Baughman B.M., Dickson B.M., Mu Y., Cui G., Mader P., Dong A., Norris J.L., Rothbart S.B., Strahl B.D. (2015). Identification of a fragment-like small molecule ligand for the methyl-lysine binding protein, 53BP1. ACS Chem. Biol..

