# Supplementary material for: Identification of a small-molecule ligand of the epigenetic reader protein Spindlin1 via a versatile screening platform
Source: Nucleic Acids Res. 2016 Feb 17;44(9):e88. doi: 10.1093/nar/gkw089 (PMC4872087; doi:10.1093/nar/gkw089)
Supplement: SUPPLEMENTARY DATA [file supp_44_9_e88__index.html]

Identification of a small-molecule ligand of the epigenetic reader protein Spindlin1 via a versatile screening platform — SUPPLEMENTARY DATA 

# Identification of a small-molecule ligand of the epigenetic reader protein Spindlin1 via a versatile screening platform

## SUPPLEMENTARY DATA

- SUPPLEMENTARY DATA
