# Supplementary material for: Identification of a small-molecule ligand of the epigenetic reader protein Spindlin1 via a versatile screening platform
Source: Nucleic Acids Res. 2016 Feb 17;44(9):e88. doi: 10.1093/nar/gkw089 (PMC4872087; doi:10.1093/nar/gkw089)
Supplement: SUPPLEMENTARY DATA [file supp_gkw089_nar-03017-met-k-2015-File010.pdf]

Supplementary Information

Identification of a Small-Molecule Ligand of the Epigenetic Reader Protein Spindlin1 via a Versatile Screening Platform

Tobias Wagner[1], Holger Greschik[2], Teresa Burgahn[1], Karin Schmidtkunz[1], Anne-Kathrin Schott[2], Joel McMillan[2], Lina Baranauskienė[3], Yan Xiong[4], Oleg Fedorov[5], Jian Jin[4], Udo Oppermann[6], Daumantas Matulis[3], Roland Schüle[2,7,8] and Manfred Jung[1,8,9*]

[1] Institute of Pharmaceutical Sciences, University of Freiburg, Freiburg, 79104, Germany.
[2] Department of Urology and Center for Clinical Research, University Freiburg Medical Center, Freiburg, 79106, Germany.
[3] Department of Biothermodynamics and Drug Design, Institute of Biotechnology, Vilnius University, Vilnius, 02241, Lithuania.
[4] Department of Structural and Chemical Biology, Department of Oncological Sciences, Department of Pharmacology and Systems Therapeutics, Icahn School of Medicine at Mount Sinai, New York, United States
[5] Structural Genomics Consortium, Nuffield Department of Clinical Medicine, University of Oxford, Target Discovery Institute (TDI), Oxford, UK
[6] Structural Genomics Consortium, Botnar Research Center, NIHR Oxford BRU, University of Oxford, Oxford OX3 7LD, UK.
[7] German Cancer Consortium (DKTK), Freiburg, Germany.
[8] BIOSS Centre of Biological Signalling Studies, University of Freiburg, 79106 Freiburg, Germany.
[9] German Cancer Research Centre (DKFZ), Heidelberg, Germany.

* To whom correspondence should be addressed. Tel: +49 761 203 4896; Fax: +49 761 203 6321; Email: manfred.jung@pharmazie.uni-freiburg.de

SYNTHESIS OF YX11-102

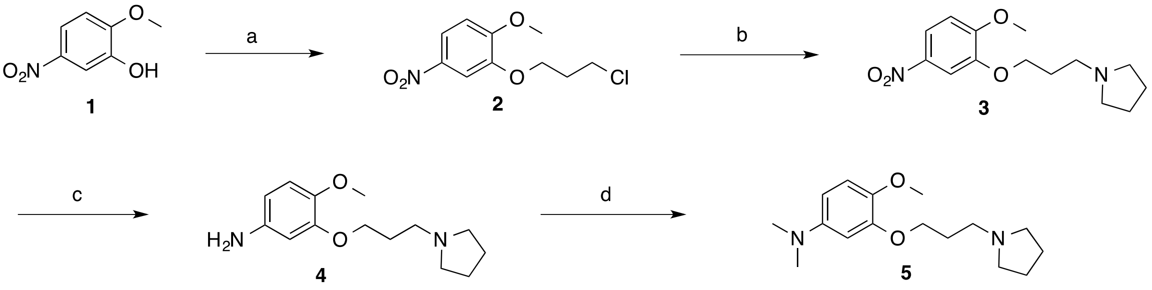

(a) 1-bromo-3-chloropropane, NaOH, *i*-PrOH, rt – 75 °C; (b) Pyrrolidine, K_2CO_3, KI, CH_3CN, reflux, 70% in 2 steps;
(c) Fe, NH_4Cl, EtOH, H_2O, reflux 60%; (d) HCHO, $NaB(CN)H_3$, HOAc, MeOH, rt, 90%;

GENERAL SYNTHESIS PROCEDURES

UPLC spectra for all compounds were acquired using a Waters 1200 Acquity-H UPLC system. Chromatography was performed on a 2.1 × 50 mm BEH-C18 1.7 µm column with water containing 0.1% formic acid as solvent A and acetonitrile containing 0.1% formic acid as solvent B at a flow rate of 0.8 mL/min. The gradient program was as follows: 3 – 100% B in 3 min. High resolution mass spectra (HRMS) data were acquired in positive ion mode using an Agilent G1969A API-TOF with an electrospray ionization (ESI) source. Nuclear Magnetic Resonance (NMR) spectra were acquired on a

Bruker DRX-600 spectrometer with 600 MHz for proton (1H NMR) and 150 MHz for carbon (13C NMR); chemical shifts are reported in ppm (δ). Preparative HPLC was performed on Agilent Prep 1200 series with UV detector set to 254 nm. Samples were injected onto a Phenomenex Luna 75 x 30 mm, 5 µm, C18 column at room temperature. The flow rate was 40 mL/min. A linear gradient was used with 10% (or 50%) of MeOH (A) in H_2O (with 0.1 % TFA) (B) to 100% of MeOH (A). HPLC was used to establish the purity of target compounds. All final compounds had > 95% purity using the HPLC methods described above.

1-(3-(2-Methoxy-5-nitrophenoxy)propyl)pyrrolidine (3). 2-Methoxy-5-nitrophenol (4.9 g, 29 mmol) was dissolved in 25 mL isopropyl alcohol. To the resulting solution was added sodium hydroxide solution (31 mL, 1 N in water, 31 mmol). After the addition was completed, the mixture was stirred for 30 minutes before being charged with 1-bromo-3-chloropropane (5.7 mL, 58 mmol) in a single portion. The resulting mixture was heated at 75 °C for 8 h before being cooled to room temperature. The product gradually precipitated from the mixture as a light yellow/tan solid. Water (200 mL) was added in a single portion and the resulting slurry was stirred for at least 30 minutes. The slurry was filtered, washed with water, and dried to provide compound **2** (6.5 g) as pale yellow solid. The crude intermediate **2** (6.5 g, 26 mmol) was dissolved in 40 mL CH_3CN. To the resulting solution were added K_2CO_3 (5.5 g, 40 mmol), KI (2.2 g, 13 mmol), and pyrrolidine (3 mL, 36 mmol) successively. This mixture was refluxed for 3 h. The solid material was filtered and wash with ethyl acetate. The filtrate was concentrated and purified by flash column chromatography on silica gel (0 – 5% MeOH (1% NH_3)/CH_2Cl_2) to afford the title compound **(3)** as brown oil (5.7 g, 70% yield in 2 steps). ^{1}H NMR (600 MHz, $CDCl_3$) δ 7.90 (dd, *J* = 9.0, 2.4 Hz, 1H), 7.77 (d, *J* = 2.4 Hz, 1H), 6.89 (d, *J* =9.0 Hz, 1H), 4.17 (t, *J* = 6.6 Hz, 2H), 3.86 (s, 3H), 2.77-2.69 (m, 2H), 2.69-2.56 (m, 4H), 2.15-2.11 (m, 2H), 1.85-1.81 (m, 4H).

4-Methoxy-3-(3-(pyrrolidin-1-yl)propoxy)aniline (4). A mixture of compound **3** (5.7 g, 20 mmol), iron dust (4.6 g, 82 mmol) and ammonium chloride (6.5 g, 122 mmol) in ethyl acetate (70 mL) and water (30 mL) was refluxed overnight. The resulting mixture was filtered and the filtrate was extracted with 5% methanol in dichloromethane (3x). The combined organic layers were dried, concentrated and purified by reverse-phase C18 column (0 – 100% methanol/water (0.1% HOAc)) to afford the title compound **4** as brown oil (4.3g, 60%).

4-Methoxy-*N,N*-dimethyl-3-(3-(pyrrolidin-1-yl)propoxy)aniline (5). Compound **4** (52 mg, 0.21 mmol) was dissolved in methanol. To the resulting solution were added formaldehyde (0.2 mL, 2.6 mmol) and $NaB(CN)H_3$ (49 mg, 0.75 mmol). The mixture was stirred at room temperature for 1 h before being concentrated and purified by HPLC to afford the title compound **5** as brown solid (95 mg, 90%). ^{1}H NMR (600 MHz, CD_3OD) δ 7.24 (d, *J* = 1.8 Hz, 1H), 7.18 (dd, *J* = 8.4, 1.8 Hz, 1H), 7.13 (d, *J* = 8.4 Hz, 1H), 4.23 (t, *J* = 5.4 Hz, 2H), 3.91 (s, 3H), 3.82-3.78 (br, 2H), 3.48 (t, *J* = 6.6 Hz, 2H), 3.25 (s, 6H), 3.18-3.12 (br, 2H). 2.32-2.29 (m, 2H), 2.24-2.17 (br, 2H), 2.11-2.03 (br, 2H); ^{13}C NMR (150 MHz, CD_3OD, 3 overlapping) δ 149.5, 148.7, 136.5, 112.4, 111.9, 104.9, 66.7, 55.3, 54.0 (2C), 53.0, 45.4 (2C), 25.3, 22.5 (2C); UPLC: 95%; t_R = 0.64 min; HRMS calcd. for $C_{16}H_{26}N_2O_2$ + H: 279.2067; found: 279.2099 [M + H]+.
